# Single-cell transcriptomics identifies new blood cell populations in *Drosophila* released at the onset of metamorphosis

**DOI:** 10.1242/dev.201767

**Published:** 2023-09-27

**Authors:** Alexander Hirschhäuser, Darius Molitor, Gabriela Salinas, Jörg Großhans, Katja Rust, Sven Bogdan

**Affiliations:** ^1^ Institute of Physiology and Pathophysiology, Department of Molecular Cell Physiology, Philipps University Marburg, Emil-Mannkopff-Strasse 2, 35037 Marburg, Germany; ^2^ NGS-Integrative Genomics Core Unit, Department of Human Genetics, University Medical Center Göttingen, Justus von Liebig Weg 11, 37077 Göttingen, Germany; ^3^ Department of Biology, Philipps University Marburg, Karl-von-Frisch-Strasse 8, 35043 Marburg, Germany

**Keywords:** *Drosophila*, Blood, Progenitor, scRNA-seq analysis, PSC, Migration, Hemocytes, Plasmatocytes, Lamellocytes

## Abstract

*Drosophila* blood cells called hemocytes form an efficient barrier against infections and tissue damage. During metamorphosis, hemocytes undergo tremendous changes in their shape and behavior, preparing them for tissue clearance. Yet, the diversity and functional plasticity of pupal blood cells have not been explored. Here, we combine single-cell transcriptomics and high-resolution microscopy to dissect the heterogeneity and plasticity of pupal hemocytes. We identified undifferentiated and specified hemocytes with different molecular signatures associated with distinct functions such as antimicrobial, antifungal immune defense, cell adhesion or secretion. Strikingly, we identified a highly migratory and immune-responsive pupal cell population expressing typical markers of the posterior signaling center (PSC), which is known to be an important niche in the larval lymph gland. PSC-like cells become restricted to the abdominal segments and are morphologically very distinct from typical *Hemolectin* (*Hml*)-positive plasmatocytes. G-TRACE lineage experiments further suggest that PSC-like cells can transdifferentiate to lamellocytes triggered by parasitoid wasp infestation. In summary, we present the first molecular description of pupal *Drosophila* blood cells, providing insights into blood cell functional diversification and plasticity during pupal metamorphosis.

## Teaser

**Summary:** The first molecular description of pupal *Drosophila* blood cells provides insights into blood cell functional diversification and plasticity during metamorphosis, a phase marked by programmed cell death and inflammatory signals.

## INTRODUCTION

The innate immune system depends on a wide array of conserved cellular and molecular strategies to mediate pathogen defense, tissue remodeling and repair. *Drosophila* is a powerful genetic model organism in which to study blood cell development and innate immunity (Banerjee et al., 2019; Evans et al., 2003; Lemaitre and Hoffmann, 2007). The fruit fly has an open circulatory system in which the heart pumps blood, the so-called hemolymph, into the body cavity circulating all organs. To fight against infections, flies evolved a large variety of defense responses that share highly conserved features with the human innate immunity (Bergman et al., 2017; Buchon et al., 2014). Similar to mammals, the first line of defense against invading pathogens and wounds in *Drosophila* relies on both a humoral response, whereby effector molecules such as antimicrobial peptides are secreted into the hemolymph, and a cellular response, in which pathogens are phagocytosed by blood cells, the so-called hemocytes (Gold and Brückner, 2015; Vlisidou and Wood, 2015). Hemocytes have been traditionally classified by their cell morphology into three different effector cells: plasmatocytes, crystal cells and lamellocytes (Rizki, 1962). Plasmatocytes, the most abundant immune cell type in flies, are professional phagocytes similar to mammalian bone marrow-derived macrophages (Gold and Brückner, 2015). Circulating and tissue-resident plasmatocytes are immediately recruited to sites of wounding and infections and mediate the major cellular immune response by phagocytosing pathogens and secreting antimicrobial and clotting factors (Evans and Wood, 2014; Sander et al., 2013). Upon injury, platelet-like crystal cells are required for the melanization of wounds, a process that involves the rapid synthesis of the black pigment melanin, which is required for wound healing and encapsulation of invading parasites (Bidla et al., 2007; Rizki and Rizki, 1959). Lamellocytes, by contrast, are rarely observed in healthy flies, but are dramatically induced in response to infection by parasitic wasps (Anderl et al., 2016).

Blood-lineage specification in flies requires a similar conserved set of transcriptional regulators and signaling pathways to those that control mammalian hematopoiesis, including the GATA factor Serpent (Srp) and the friend of GATA (FOG) transcriptional co-factor U-shaped (Crozatier and Vincent, 2011). *Drosophila* hematopoiesis occurs in two spatially and temporally distinct phases with clear parallels with the mammalian process (Crozatier and Meister, 2007). Blood cells initially derive from the head mesoderm of the developing embryo and give rise to both plasmatocytes and crystal cells. These cells colonize and self-renew in segmentally repeated epidermal–muscular niches in larvae (Crozatier et al., 2004; Makhijani et al., 2011). The hematopoietic pockets provide both an attractive and trophic microenvironment, promoting proliferation of the initial 600-700 embryonic cells to about 9000-10,000 cells per third-instar in the differentiated state (Petraki et al., 2015). The second wave of hematopoiesis in flies occurs post-embryonically in the lymph gland of larvae, a specialized hematopoietic organ of mesoderm origin, which is arranged in multiple paired lobes along the anterior part of the dorsal aorta (Lanot et al., 2001). The lymph gland harbors progenitors, as well as differentiating and mature blood cells within distinct zones: the cortical zone (CZ) with mature hemocytes, the medullary zone (MZ) with progenitors and the posterior signaling center (PSC). The PSC is thought to function as a niche to control the differentiation of all three effector types from progenitors (Jung et al., 2005; Lebestky et al., 2003). The PSC comprises a small cluster of about 30-40 cells and is mitotically inactive in the mature lymph gland. These cells are marked by high levels of a conserved member of the Early B-cell Factor (EBF) family of transcription factors, Knot (Kn; also known as Collier, Col), which controls PSC specification and cell numbers, expression of the homeobox protein Antennapedia (Antp) and absence of Ultrabithorax (Ubx) expression (Krzemień et al., 2007, 2010; Mandal et al., 2007; Boulet et al., 2021; Rodrigues et al., 2021; Kanwal et al., 2021). The PSC was initially suggested to control the balance between undifferentiated precursor cells and differentiating hemocytes (Krzemień et al., 2007; Mandal et al., 2007). However, more recent studies further revealed a more complex role of the PSC in regulating blood cell differentiation, rather than maintaining core progenitors in lymph gland homeostasis (Benmimoun et al., 2015a,b; Oyallon et al., 2016; Baldeosingh et al., 2018). Supporting this notion, genetic ablation of the PSC did not markedly affect the blood cell progenitor population, but abrogated lamellocyte differentiation in response to parasitoid wasp infection (Benmimoun et al., 2015b; Oyallon et al., 2016). Interestingly, PSC-like cells have been identified by single-cell RNA sequencing (scRNA-seq) among circulating blood cells of larvae and adults (Cattenoz et al., 2020; Tattikota et al., 2020; Fu et al., 2020; Li et al., 2022). In adults, these PSC-like cells can proliferate and differentiate into plasmatocytes in response to bacterial infection (Boulet et al., 2021).

Under normal conditions, cell progenitors in the lymph gland give rise to plasmatocytes and crystal cells. Both effector cell types are released into the circulation as the lymph gland dissociates at the onset of metamorphosis (Grigorian et al., 2011). Thus, with the onset of pupation, both embryo and lymph gland-derived blood cells mix and populate the pupa. Recent advances in scRNA technologies have further revealed a much larger diversity of the hemocytes in the *Drosophila* larva as well as in the lymph gland and adult fly (Cattenoz et al., 2020; Cho et al., 2020; Fu et al., 2020; Girard et al., 2021; Leitão et al., 2020; Li et al., 2022; Tattikota et al., 2020). Comparative analysis of these datasets showed important differences in the molecular signatures between hemocytes during embryogenesis and larval stages and further suggested increased complexity of hemocyte populations in metamorphosis and in adult flies (Cattenoz et al., 2021; Hultmark and Andó, 2022; Li et al., 2022; Liegeois and Ferrandon, 2020). Metamorphosis is accompanied by dramatic and systemic physiological changes that require integrating the innate immune system. In response to ecdysone, hemocytes rapidly upregulate cell motility and phagocytosis of apoptotic debris, and acquire the ability to relocate by chemotaxis to areas of tissue damage (Regan et al., 2013; Sampson et al., 2013; Sander et al., 2013). Bulk RNA-seq gene expression analysis recently uncovered thousands of genes that are differentially expressed in pupal hemocytes compared with larvae (Lehne et al., 2022). However, whether this striking differential expression pattern of pupal hemocytes also reflects the differentiation of new hemocyte subtypes or cell types has yet to be addressed. Insights into heterogeneity of these activated hemocytes would, however, also shed light on how tissue clearance is regulated during development.

Here, we employ scRNA-seq technology to characterize the molecular signatures of pupal hemocyte subpopulations in the early stages of metamorphosis. Our data reveal the presence of different undifferentiated and specified hemocytes with distinct transcriptomic signatures associated with distinct functions, such as antimicrobial, antifungal immune defense, cell adhesion or secretion. Cross-stage dataset analysis provides evidence that some pupal hemocyte populations display distinct transcriptomic profiles, suggesting specific functions during metamorphosis. Among the pupal blood cell types, we identified a highly migratory and immune-responsive PSC-like cell cluster that persists into the adult fly. Lineage-tracing experiments suggest that these PSC-like cells are plastic and able to differentiate to lamellocytes in response to wasp infestation.

## RESULTS

### *Drosophila* pupal hemocytes display remarkable cellular heterogeneity

**Fig. 1.**
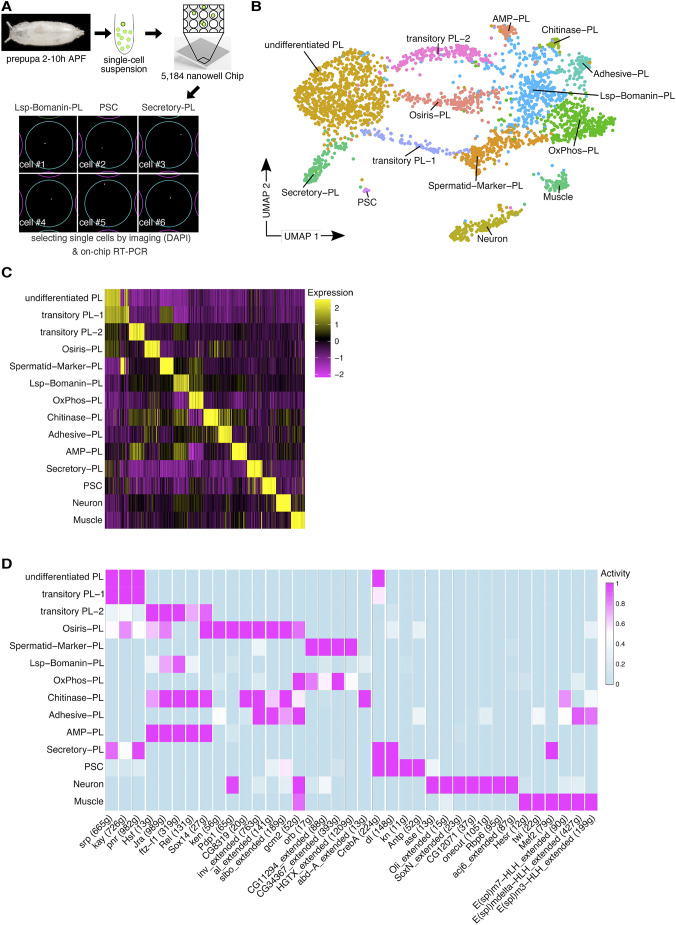
**scRNA-seq analysis of pupal *Drosophila* hemocytes reveals a remarkable cellular heterogeneity.** (A) Workflow for the full-length SMART scRNA-Seq approach using the ICELL8 platform. Only wells containing single cells were selected using the CellSelect Software prior to the on-chip RT-reaction, library preparation and sequencing. Example images of Lsp-Bomanin-PL, PSC and Secretory-PL cells are shown. (B) UMAP plot of 2811 high-quality cells in 14 transcriptomically distinct clusters. (C) Heatmap of the top 50 differentially regulated genes per cluster. (D) Transcription factor activity of selected transcription factors identified by SCENIC (Aibar et al., 2017).

Recent comparative scRNA-seq analyses revealed cellular heterogeneity in the molecular signatures of *Drosophila* embryonic and larval hemocytes (Cattenoz et al., 2021, 2020; Fu et al., 2020; Girard et al., 2021; Hultmark and Andó, 2022; Leitão et al., 2020; Tattikota et al., 2020). Both embryo and lymph gland-released hemocytes mix at the onset of pupariation and persist into adulthood. Our recent bulk RNA analysis revealed a highly differential expression profile in pupal hemocytes compared with larvae (Lehne et al., 2022). To analyze the cellular heterogeneity of the total blood cells in *Drosophila* early pupa at stage 2-10 h after puparium formation (APF), we applied single-cell RNA SMART-Seq technology on the ICELL8 system (Fig. 1A; Shomroni et al., 2022). A total of 2811 high-quality cells from three replicates were used for subsequent cluster analyses (Fig. S1A). Using hierarchical clustering, we identified a total of 15 cell clusters. Two of these clusters revealed a large overlap of expressed markers and differential gene expression analysis failed to identify markers to discriminate between these clusters. We decided to merge these two clusters (resulting in the cluster ‘undifferentiated PL’). For the resulting 14 clusters, we identified unique sets of differentially expressed genes (DEGs; Fig. 1B,C, Tables S1, S2). All three datasets contributed to each of these 14 clusters, although we noted unequal contribution in some cases (Fig. S1B-D). To determine the cellular identity of each cluster, we analyzed DEGs (Tables S3-S16) as well as transcription factor activity. We used SCENIC (Aibar et al., 2017) to assess the expression of the transcription factors and corresponding target genes (Fig. 1D, Tables S17, S18). The designation of each cluster corresponds to distinct marker genes or specific biological features (see subsequent GO term analysis, Fig. 3H). Twelve cell clusters showed a significant expression of the pan-hemocyte marker *Hemese* (*He*), but also known pan-plasmatocyte markers such as genes encoding the phagocytosis receptor NimC1 (antigen marker P1) and the scavenger receptor Croquemort (Crq) (Fig. S1E). Two clusters contained non-hemocytes expressing muscle- or neuron-specific DEGs and displayed activity of transcription factors specific for the respective cell type (Fig. S2A-L). We did not observe a population expressing lamellocyte markers, as expected for non-infested animals (Fig. S1E). Remarkably, we also did not identify a distinct crystal cell cluster in our dataset differentially expressing *lozenge* (*lz*) or *hindsight* (*hnt*; *pebbled*, *peb*) (Fig. S1E). Accordingly, we only found very few Hnt-positive hemocytes isolated from pupal hemolymph (0.37%, *n*=545) compared with larval preparations (3.48%, *n*=517). This suggests that crystal cells represent either a rare cell type in *Drosophila* pupae and adults, as recently reported (Ghosh et al., 2015), or that many cells may have been lost as a result of the sensitive nature of crystal cells, which tend to burst after bleeding (Bidla et al., 2007; Hultmark and Andó, 2022).

Using a bulk RNA-sequencing approach, we previously identified several cytoskeleton and cell motility genes upregulated in pupal hemocytes (Lehne et al., 2022). Among the top 50 of these genes, 37 genes showed cluster specific expression profiles (Fig. S2M). Many of these genes were highly expressed in undifferentiated PL, the most abundant cell cluster we identified. Another subset of these genes showed differential expression in OxPhos-PL, Adhesive-PL and Chitinase-PL, suggesting that these populations change transcription profiles with the onset of pupariation to become motile. To compare pupal hemocyte populations with hemocyte clusters of other developmental stages, we examined cell type-specific marker genes identified in other scRNA-seq studies (Cattenoz et al., 2020; Cho et al., 2020; Fu et al., 2020; Girard et al., 2021; Leitão et al., 2020; Li et al., 2022; Tattikota et al., 2020). Most of the pupal hemocyte populations did indeed express marker genes corresponding to described larval, lymph gland and/or adult hemocyte populations (Fig. S3A, Table S1). These similarities are presented in more detail below.

### Undifferentiated plasmatocytes are the largest subgroup in the prepupa

**Fig. 2.**
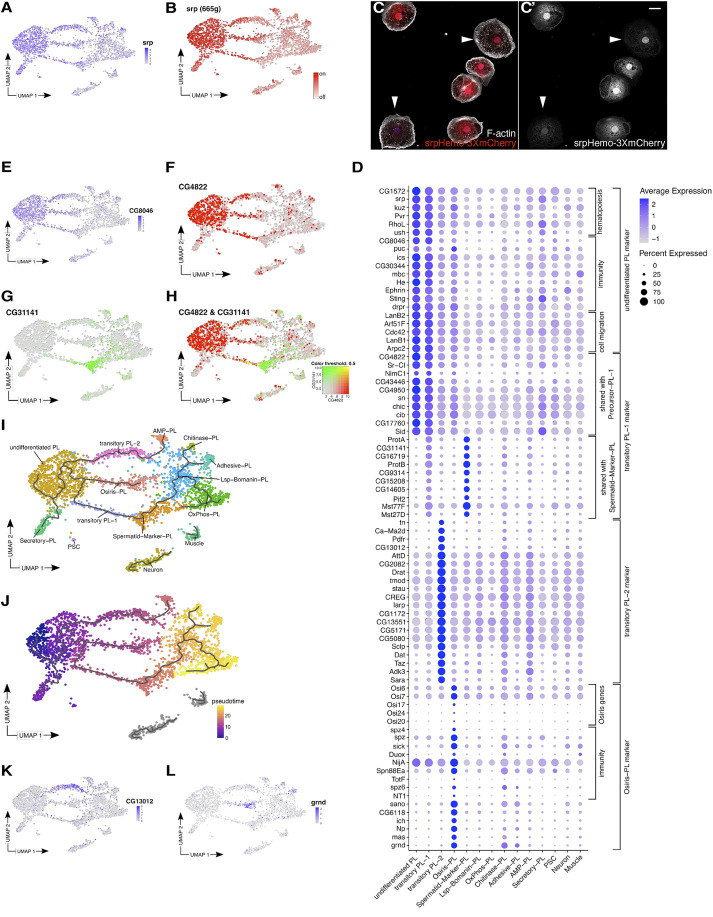
**Identification of several undifferentiated plasmatocyte populations.** (A,B) UMAP plots. *srp* is highly expressed (A) and active (B) in several clusters. (C,C′) Confocal images of pupal hemocytes expressing srpHemo-3xmCherry; white arrowheads highlight low-expressing cells. The data show representative images replicated in three independent experiments. Scale bar: 10 µm. (D) Dotplot showing the average expression and percentage expression per cluster for marker genes of clusters with high *srp* expression: undifferentiated PL, transitory PL-1, PL-2 and Osiris-PL. (E-I) Expression of transitory PL-1 markers on UMAP plots. (E) Expression of *CG8046*, an undifferentiated PL marker. (F) *CG4822* expression is also detected in undifferentiated PL cells. (G) *CG31141* expression is also detected in the Spermatid-Marker-PL cluster. (H) Combined expression of *CG4822* and *CG31141* is only detected in the transitory PL-1 cluster. (I,J) Monocle3 pseudotime analysis. (I) Pseudotime trajectory on a UMAP plot. (J) UMAP plot with pseudotime trajectory and cells colored based on pseudotime. (K,L) Expression of marker genes on UMAP plots. (K) *CG13012*, identified as a marker for the transitory PL-2 cluster. (L) *grindelwald* (*grnd*), identified as a marker for the Osiris-PL cluster.

The GATA transcription factor Serpent (Srp), which is required for prohemocyte specification in the embryo (Rehorn et al., 1996), was previously proposed as progenitor marker in the adult fly (Ghosh et al., 2015); however, the presence of a proliferating hemocyte precursor stage in adults has been refuted more recently (Sanchez Bosch et al., 2019). In the pupa, we found several cell clusters with higher levels of *srp* expression and *srp* activity (SCENIC), including undifferentiated PL, transitory PL1-2, Osiris-PL and Secretory-PL (Figs 1D and 2A,B). Expression analysis of a *srp* Gal4 promoter trap that we expect to reflect *srp* mRNA levels confirmed that *srp* expression is heterogeneous in hemocytes (Fig. 2C). Undifferentiated-PL displayed the highest levels of *srp* expression and transcription factor activity (Fig. 2A,B,D). This cluster corresponds to a larval plasmatocyte population (PL-0, Cattenoz et al., 2020; PLASM1, Leitão et al., 2020) and/or a lymph gland plasmatocyte population (PM1-2, Cho et al., 2020; PL2, Girard et al., 2021) as well as an adult plasmatocyte subtype (Plasmatocytes nAChRalpha3 and *trol* high, Li et al., 2022; Fig. S3A, Table S1).

Markers expressed in undifferentiated PL were also detected in other clusters with high *srp* expression, such as the transitory PL-1 (Fig. 2A,B,D,E). However, this cluster differed from undifferentiated PL by sharing markers with the low *srp*-expressing cluster Spermatid-Marker-PL (Fig. 2D,F-H), suggesting that transitory PL-1 could be a transition state towards a more specified plasmatocyte fate. To test this hypothesis, we applied pseudotime analysis using monocle3 (Trapnell et al., 2014). This method orders cells based on stepwise transcriptomic changes with the most undifferentiated state at one end and more differentiated states at the other end. Indeed, the pseudotime analysis located the undifferentiated PL cluster at one end of the trajectory, suggesting that these are the most undifferentiated plasmatocytes in the pupa (Fig. 2I,J). Transitory PL-1 located between undifferentiated PL and Spermatid-Marker-PL, confirming that it is likely a transition state during plasmatocyte specification. We also found transitory PL-2 to be a transition state towards the more differentiated AMP-PL state.

**Fig. 3.**
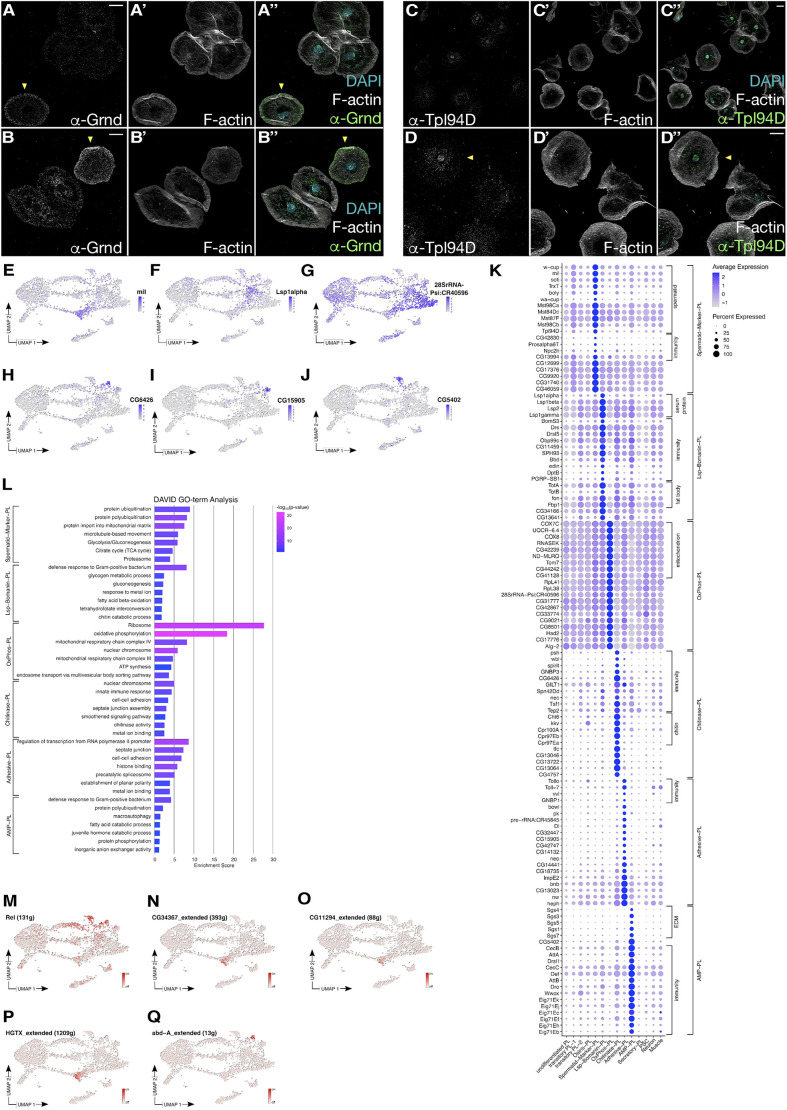
**Identification of effector cells with distinct molecular signatures.** (A-B″) Confocal images of pupal hemocytes stained with an anti-Grindelwald (Grnd) antibody. Yellow arrowheads indicate plasmatocytes with marked Grnd expression. Scale bars: 10 µm. (C-D″) Confocal images of pupal hemocytes stained with an anti-Tlp94D antibody. Yellow arrowheads indicate plasmatocytes with marked Grnd expression. Scale bars: 10 µm. (E-J) Expression of selected markers on UMAP plots. (E) *mil* is a marker for the Spermatid-Marker-PL cluster. (F) *Lsp1α* is expressed in Lsp-Bomanin-PL cells. (G) *28SrRNA-Psi:CR40596* is highly expressed in the OxPhos-PL cluster. (H) *CG6426* is a marker for the Adhesive-PL cluster. (I) *CG15905* marks the Adhesive-PL cluster. (J) *CG5402* marks the AMP-PL cluster. (K) Dotplot of selected marker genes with average expression and percent expression per cluster for the following clusters: Spermatid-Marker-PL, Lsp-Bomanin-PL, OxPhos-PL, Chitinase-PL, Adhesive-PL and AMP-PL. ECM, extracellular matrix. (L) Representative terms of the top seven annotation clusters identified with DAVID 2021 GO term analysis of DEGs of the clusters Spermatid-Marker-PL, Lsp-Bomanin-PL, OxPhos-PL, Chitinase-PL, Adhesive-PL and AMP-PL. The bar length reflects the enrichment score of the annotation cluster and is colored based on the *P*-value of the GO term. (M-Q) Activity of transcription factors on UMAP plots identified with SCENIC. (M) *Rel* is highly active in Precursor-PL-3, AMP-PL and Chitinase-PL clusters. (N-P) *CG34367* (N), *CG11294* (O) and *HGTX* (P) are active in the Spermatid-Marker-PL cluster. (Q) *abd-A* is active in the Chitinase-PL cluster. The data show representative images replicated in three independent experiments.

Despite overlapping marker genes, transitory PL-2 and Osiris-PL could be distinguished from undifferentiated PL by the expression of cluster-specific markers, transcription factor activity and distinct gene ontology (GO) terms (Fig. 2D,K,L; Fig. S3B-F). The Osiris-PL cluster was eponymously characterized by the expression of members of the insect-specific gene family Osiris, encoding putative transmembrane proteins linked to the development of resistance against a range of different plant and fungi toxins (Fig. 2D; Lanno et al., 2019; Smith et al., 2018; Trienens et al., 2017). In addition, Osiris-PL expressed many genes involved in immune response and inflammation, including genes encoding the Tumor Necrosis Factor (TNF) receptor Grindelwald (Grnd) and Ninjurin A (NijA), a conserved adhesion molecule that mediates plasma membrane rupture in cell death (Degen et al., 2023; Fig. 2D,L). Immunostaining with a specific anti-Grnd antibody confirmed the differential expression of Grnd in a subset of pupal, but not larval, plasmatocytes (5.5% positive cells, *n*=488; Fig. 3A-B″). The Osiris-PL cluster also showed a characteristic increased activity of *invected* (*inv*), a transcription factor that is specifically required for anti-fungal defense (Fig. S3D) (Jin et al., 2008). The Osiris-PL were also characterized by increased activities of transcription factors controlling a subset of JAK/STAT pathway target genes, such as *ken and barbie* (Ekengren and Hultmark, 2001), which in turn regulates *slow border cells* (*slbo*) gene expression (Fig. S3E). Interestingly, Osiris-PL did not express a gene profile correlating to larval, lymph gland or adult cell populations, suggesting that it might be a new cell population of specialized hemocytes specific for metamorphosis (see Fig. S3A, Table S1).

### Identification of specialized plasmatocyte subtypes with distinct molecular signatures

We identified six types of *srp* low-expressing hemocyte populations with distinct markers, suggesting that these are specialized immune cells (Fig. 3E-Q). Many of these clusters were active for *Relish*, a well-known transcription factor that acts downstream of the immune deficiency (IMD) pathway, regulating antibacterial responses (Fig. 3M; Imler and Hoffmann, 2000). Among them, we identified Spermatid-Marker-PL, Lsp-Bomanin-PL, OxPhos-PL, Chitinase-PL, Adhesive-PL and AMP-PL clusters.

The Spermatid-Marker-PL cluster expressed immunity genes and a high number of genes that have previously been identified in spermatids, such as *Transition protein-like 94D* (*Tpl94D*), which encodes an HMG-box domain protein (Rathke et al., 2007). Immunostaining using an anti-Tpl94D antibody labeled a subset of hemocyte nuclei accounting for about 10% of total blood cells (*n*=110 cells) isolated from pupa (Fig. 3C-D″). A cluster with similar marker gene expression has been identified among adult hemocytes (compare with Fig. S3A, Table S1; Li et al., 2022). The cluster was further characterized by activity of the transcription factors CG34367, CG11294 and HGTX (Fig. 3N-P). Among the immunity genes, we also found a member encoding the conserved Niemann-Pick type C protein family (Npc2h) protein (Fig. 3K), which is involved in immune signaling, particularly in the recognition of pathogen-related products such as lipopolysaccharides, lipid A, peptidoglycan and lipoteichoic acid (Shi et al., 2012). Furthermore, we identified *milkah* (*mil*) as cluster-specific marker (Fig. 3K), encoding a conserved nucleosome assembly factor of the Nap family involved in spermatogenesis and long-term memory formation (Dubnau et al., 2003; Kimura, 2013).

The Lsp-Bomanin-PL cluster expresses several genes encoding larval serum proteins such as Lsp1α and its receptor Fbp1 (Fig. 3F,K). The larval plasmatocyte cluster PL-Lsp (Cattenoz et al., 2020) and Lsp+ (Fu et al., 2020) displayed a corresponding expression profile (compare with Fig. S3A; Table S1). Other genes expressed by Lsp-Bomanin-PL included Bomanin genes such as *BomS3* and genes encoding secreted anti-fungal and antibacterial peptides such as *Drosomycin* (*Drs*) or *Drosomycin-like 5* (*Drsl5*) (Fig. 3K). GO term analysis revealed an enrichment for genes important for the defense response to Gram-positive bacteria (Fig. 3L). Interestingly, these cells also expressed several genes for which the products are secreted from the fat body, including stress-induced humoral factors such as *Turandot A* and *B* (*TotA*, *TotB*) and the coagulation factor-encoding *fondue* (*fon*; Fig. 3K; Bajzek et al., 2012; Ekengren and Hultmark, 2001).

The OxPhos-PL cluster expressed fewer genes than other plasmatocyte clusters (Fig. S4A). However, the number of detected unique molecular identifiers (UMIs), as well as the percentage of mitochondrial genes, were similar (Fig. S4B,C), leading us to keep this cluster as high-quality cells. A cell type with a similar expression pattern was present among larval circulating hemocytes (PM12, Tattikota et al., 2020; Fig. S3A, Table S1). OxPhos-PL showed a striking increased expression of several genes involved in mitochondrial oxidative phosphorylation system (Fig. 3K,L). This cluster also specifically expressed genes encoding several ribosomal proteins (Fig. 3K), which might reflect an important cross-regulatory mechanism between mitochondrial energy production and increased ribosomal assembly and translation, as recently described for macrophage tissue invasion in the *Drosophila* embryo (Emtenani et al., 2022).

The Chitinase-PL cluster showed *abd-A* activity and expressed high levels of immunity relevant genes, such as *persephone* (*psh*) and *spirit*, which encode serine proteases, but also diverse regulators of the Toll signaling pathway, including *windbeutel* (*wbl*), *Gram-negative bacteria binding protein 3* (*GNBP3*), *Gamma-interferon-inducible lysosomal thiol reductase 1* (*GILT1*), *Transferrin 1* (*Tsf1*) and *Thioester-containing protein 2* (*Tep2*), which mediate the cellular immune response to bacteria (Fig. 3K,L,Q). The characteristic high expression of *Cht6* chitinase, an evolutionarily conserved enzyme involved in ecdysis, organization of the exoskeletal barrier, but also in immune defense in vertebrates, prompted us to name this cluster accordingly (Fig. 3K). Recent studies further revealed that the functions of chitinases are not solely to catalyze the hydrolysis of chitin producing pathogens, but also include a crucial role in bacterial infections and inflammatory diseases (Di Rosa et al., 2016).

The Chitinase-PL and the Adhesive-PL clusters expressed several genes implicated in cell–cell adhesion, which was more prominent for Adhesive-PL, prompting us to name this cluster accordingly (Fig. 3K, Fig. S4D). The expressed markers ranged from cadherin-encoding genes such as *shotgun* (*shg*), *echinoid* (*ed*) *Cad87A* and *fat* (*ft*) to *Fasciclin 3* (*Fas3*), *prickle* (*pk*) and *piopio* (*pio*), which are known to be involved in epithelial cell polarity. Interestingly, the Adhesive-PL cluster was characterized by low levels of *atilla* and *cheerio* expression (compared with other clusters, Fig. S1E), and thus might represent an intermediate state with the potential to transdifferentiate into terminally differentiated lamellocytes, as recently described (Anderl et al., 2016; Csordás et al., 2021).

The AMP-PL cluster expressed a high number of immunity genes and our GO term analysis revealed an enrichment in genes involved in defense to Gram-positive bacteria (Fig. 3K,L). Among the immunity genes, we identified a large repertoire of AMP (Antimicrobial peptide)-encoding genes, including *Attacin-A*, *Attacin-B*, *Drosomycin-like 1*, *Drosomycin-like 6*, *Cecropin A* (*CecropinA1*), *Cecropin B* and *Drosocin* (Fig. 3K). Several of these genes have been identified as markers of larval (PL-AMP; Cattenoz et al., 2020) and adult plasmatocytes (plasmatocytes Cec and other immunity genes high; Li et al., 2022; Fig. S3A, Table S1). However, the AMP-PL cluster also showed an increased number of ecdysone-inducible genes involved in pupal morphogenesis (*Eig71Ek*, *Eig71Ej*, *Eig71Ec*, *Eig71Ef*, *Eig71Eh*, *Eig71Eb*; Fig. 3K; Wright et al., 1996). The AMP-PL cluster also expressed several genes encoding extracellular matrix components, including salivary gland secretion (Sgs) proteins, which are also induced by ecdysone (Fig. 3K; Lehmann, 1996).

**Fig. 4.**
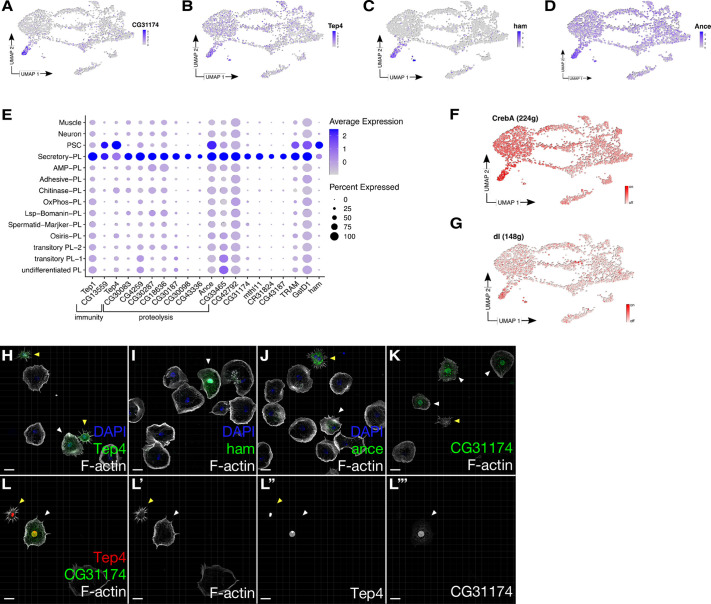
**Secretory-PL are transcriptomically distinct from other pupal blood cell types.** (A-D) UMAP plots showing the expression of Secretory-PL markers. (A) *CG31174* is specific for Secretory-PL. (B-D) *Tep4* (B), *ham* (C) and *Ance* (D) expression can also be detected in PSC cells. (E) Average expression and percentage expression of Secretory-PL markers on a dotplot. Note the importance of many genes in immunity or their function in proteolysis. (F,G) Activity of *CrebA* (F) and *dl* (G), transcription factors that are highly active in the Secretory-PL cluster on UMAP plots as identified by SCENIC. (H-L‴) Maximum intensity projections of confocal images of pupal hemocytes expressing GFP; cells with plasmatocyte-like cell shape are marked by white arrowheads; small spiky cells with filopodial protrusions are marked by yellow arrowheads; Alexa 568-labeled phalloidin was used to stain the actin cytoskeleton. (H) Tep4-Gal4. (I) Ham-Gal4. (J) Ance^mimic^-GFP. (K) CG31174^mimic^-GFP. (L-L‴) Small spiky cells are not CG31174 positive. CG31174^mimic^-GFP co-expressing *Tep4*-Gal4-nls-mCherry. Small spiky cells are marked by a yellow arrowhead and plasmatocyte-like shaped cells co-expressing both markers are marked by a white arrowhead. The data show representative images replicated in three independent experiments. Scale bars: 10 µm.

The Secretory-PL cluster was one of the *srp*-high expressing clusters but without any descending clusters (Fig. 2A,B,I,J). The Secretory-PL cluster expressed some markers identified in circulating larval blood cells (PL-Pcd, Cattenoz et al., 2020; thanacytes, Fu et al., 2020; Fig. S3A, Table S1), suggesting that these cells may be of embryonic origin. This cluster expressed many genes encoding proteins involved in proteolysis with annotated serine-type endopeptidase activity (*CG30098*, *CG31174*, *CG30083*, *CG18636*, *CG14088*) and the intracellular membrane system as well as protein export, inspiring us to name this cluster Secretory-PL (Fig. 4A,E, Fig. S5A). SCENIC further identified high activity of the cAMP response element binding (CREB) protein CrebA, a transcription factor regulating components of the secretory pathway (Johnson et al., 2020; Fig. 4F). In addition, target genes of *dorsal*, encoding the executive transcription factor downstream of Toll signaling pathway, were highly expressed (Fig. 4G). The Secretory-PL cluster shared some marker genes with another cluster that we later identified as PSC-like cells (Fig. 4B-E). Shared markers included the genes *Tep4*, *Ance* and *ham* (Fig. 4B-D). Using Gal4-enhancer and GFP-exon traps for these genes, we identified two morphologically distinct cell types: plasmatocyte-shaped cells and small spiky cells (Fig. 4H-J). In contrast, *CG31174* was only detected in cells with a normal plasmatocyte morphology (Fig. 4K). To confirm that these morphologically different cells are indeed distinct cell types, we co-labeled CG31174 with Tep4 and, indeed, whereas plasmatocyte-shaped cells co-expressed both markers, small spiky cells were never CG31174 positive. Thus, we conclude that Secretory-PL are plasmatocytes with a classical cellular morphology (Fig. 4L-L‴). *CG31174* has been recently shown to be expressed in crystal cells (Fu et al., 2020). We did not detect any classical crystal cell markers in the Secretory-PL cluster (Fig. S1E) and neither larval nor pupal CG31174 cells with plasmatocyte morphology expressed the crystal cell marker Hnt. As mentioned above, we detected a very low number of crystal cells in pupal bleeds. Among six replicates of pupal bleeds in which we stained CG31174 cells with anti-Hnt antibody, we identified one anti-Hnt/CG31174 double-positive cell from a total of 3000 isolated cells. This cell displayed crystal cell morphology. In larval bleeds, CG31174 cells with crystal cell morphology and positive for Hnt were more abundant. Thus, CG31174 we conclude that is expressed in crystal cells, as recently reported (Fu et al., 2020). However, the pupal CG31174-positive cell cluster represents a plasmatocyte cell type distinct from crystal cells. To confirm that Secretory-PL cells are distinct from crystal cells, we made use of datasets available in public single-cell databases from various stages (Cattenoz et al., 2020; Cho et al., 2020; Li et al., 2022), merged and batch-corrected the data with our dataset (Fig. S5B). Whereas larval and adult crystal cells located on the same region in the Uniform Manifold Approximation and Projection (UMAP) plot, the Secretory-PL cluster located differently, confirming that Secretory-PL cells differ from crystal cells (Fig. S5C-F).

### Identification of a pupal cell population expressing typical markers of the PSC

**Fig. 5.**
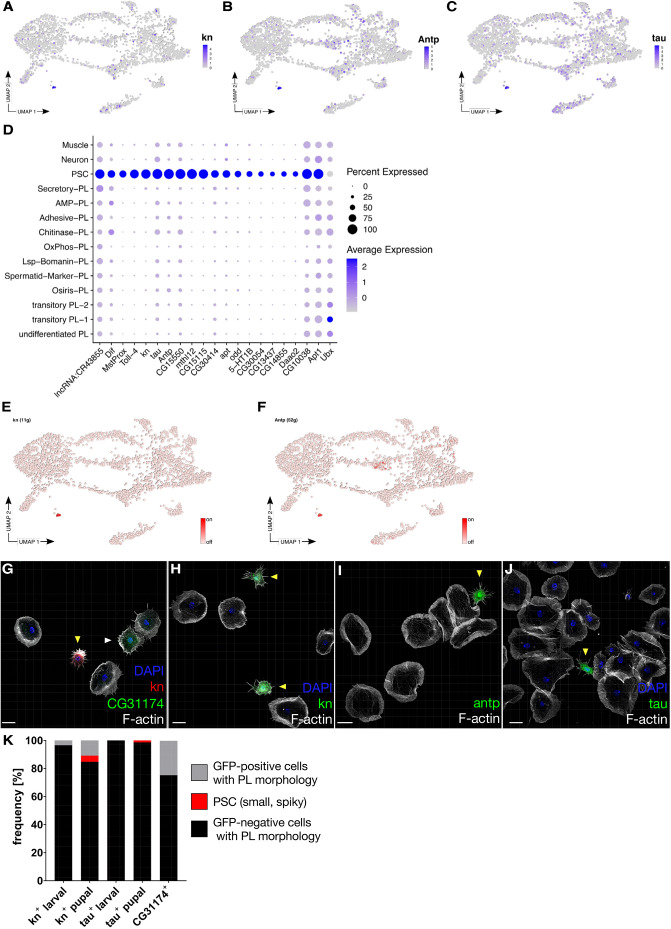
**Identification of individual PSC cells in pupae.** (A-C) Expression of the PSC markers *kn* (A), *Antp* (B) and *tau* (C) on UMAP plots. (D) Dotplot with average expression and percentage of expression of PSC markers. (E,F) Activity of transcription factors *kn* (E) and *Antp* (F) on UMAP plots. (G-J) Maximum intensity projections of confocal images of the actin cytoskeleton in pupal macrophages; phalloidin (white) was used to stain the actin cytoskeleton and DAPI for the nuclei (blue). The data show representative images replicated in three independent experiments. (G) PSC cells and Secretory-PL cells represent different cell types. CG31174 mimic-GFP co-expressing *kn*-Gal4-mCherry. PSC cells are marked by a yellow arrowhead and CG31174-positive cells are marked by a white arrowhead. (H) *kn*-Gal4 driving UAS-GFP. (I) *Antp*-Gal4 driving UAS-GFP. (J) *tau*-Gal4 driving UAS-GFP. (K) Quantification of GFP-positive spiky PSC cells, GFP-positive and GFP-negative cells with plasmatocyte morphology frequency represented by *kn*, pupal (*n*=638; GFP-positive=4.4% spiky and 10.8% round); *kn*, larval (*n*=351; GFP-positive: 0% spiky, 3.4% round); *tau*, pupal (*n*=464; GFP-positive: 1.4% spiky and 0% round). No *tau*-positive cells were found in larval bleeds. CG31174: *n*=359; GFP-positive=24.5%; GFP-negative=75%. Cells were obtained from six individual experiments. Scale bars: 10 µm.

In addition to the major plasmatocyte clusters, our scRNA-seq also identified a small cell cluster with a very unique set of markers, including the early hemocyte marker *srp*, but without detectable levels of more mature hemocyte markers such as *He*, *Pxn* and *crq* (Fig. 2A,D, Fig. S1E, GO term analysis in Fig. S6A). Remarkably, this cell cluster expressed striking markers of the PSC present in the lymph gland, such as *knot* (*kn*; Makki et al., 2010) and the recently identified new PSC marker *tau* (Cho et al., 2020; Fig. 5A-D). *kn* is also expressed in a subset of cells in the posterior lymph gland, which have yet to be subjected to scRNA-seq (Benmimoun et al., 2015b; Kanwal et al., 2021; Rodrigues et al., 2021). However, in contrast to posterior lymph gland cells, PSC cells express Antp but do not express Ubx (Benmimoun et al., 2015b; Boulet et al., 2021; Kanwal et al., 2021; Rodrigues et al., 2021). In agreement with a lymph gland PSC origin, the pupal PSC-like cell cluster expressed *kn*, *tau* and *Antp*, but not *Ubx* (Fig. 5D). Both *Antp* and *kn* were also active in the PSC cluster according to SCENIC analysis (Fig. 5E,F). We queried datasets containing larval cells, lymph gland and adult hemocytes and found a corresponding cluster in each stage (Fig. S6B-D; Table S1; Cattenoz et al., 2020; Cho et al., 2020; Fu et al., 2020; Girard et al., 2021; Li et al., 2022). PSC-like cells can be found in the lymph gland and in adult tissue, suggesting that these cells persist throughout life (Boulet et al., 2021; Li et al., 2022). We characterized PSC-like cells morphologically in more detail. Using transgenic Gal4-enhancer and GFP-exon trap fly lines, we found that the PSC markers *kn*, *Antp* and *tau* labeled small spiky cells reminiscent of those cells identified by shared markers (*Tep4*, *ham*, *Ance*) as Secretory-PL (Figs 4H-L‴ and 5G-J). This cellular morphology is reminiscent of the fusiform PSC-like cells identified recently in adults (Boulet et al., 2021). Co-staining for CG31174 further confirmed that PSC cells are morphologically distinct from Secretory-PL cells (Fig. 5G).

In contrast to *tau*, *kn* also labeled cells with a more plasmatocyte-like round morphology (Fig. 5H). However, this population of *kn*-positive plasmatocytes was less abundant than plasmatocytes labeled by the Secretory-PL marker *CG31174*, suggesting that they are distinct cell populations (Fig. 5K). *kn*-positive cells made up 15.2% of the pupal hemocytes (*n*=636), but only 4.4% of the cells exhibited a spiky shape (Fig. 5K). Interestingly, we also found *kn*-positive cells in larval bleed (3.4%, *n*=351; Fig. 5K). However, all labeled larval cells showed a plasmatocyte-like round morphology suggesting that these morphologically different cells have distinct origins (Fig. 5K). Given that *kn* is not only expressed in the lymph gland PSC but also in the MZ and posterior lobes (Benmimoun et al., 2015b; Boulet et al., 2021; Kanwal et al., 2021; Rodrigues et al., 2021), additional *kn-*positive cells with plasmatocyte morphology might derive from the MZ or posterior lobes. In contrast, the *tau*-Gal4 driver exclusively labeled small spiky cells from pupal bleeds but in a lower overall number of cells (1.4%, *n*=573; Fig. 5J,K). No *tau*-positive hemocytes could be found in larva (*n*=505; Fig. 5K).

### The PSC cell cluster can differentiate into lamellocytes upon wasp infestation

**Fig. 6.**
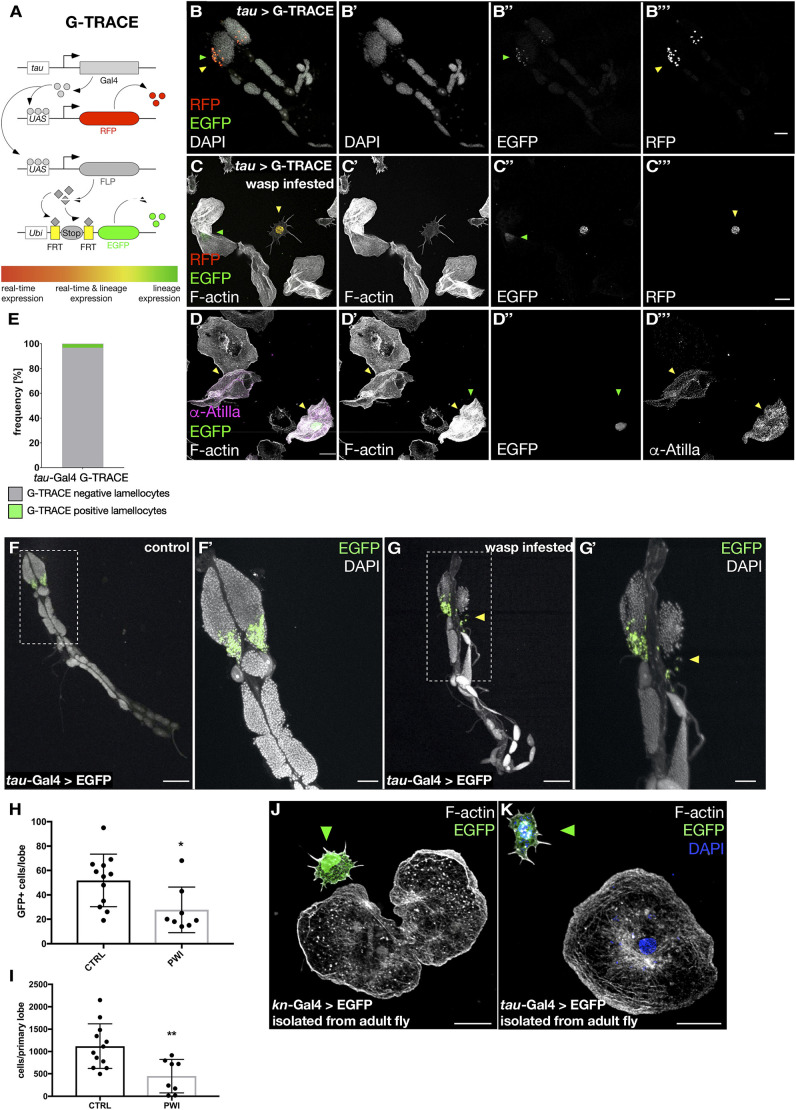
**PSC cells can differentiate into lamellocytes upon wasp infestation.** (A) Schematic overview of the G-TRACE system. (B-B‴) Activation of G-TRACE with *tau*-Gal4 results in labeling of PCS cells of third instar lymph gland. No expression can be observed in other cells outside the PSC region. (C-D‴) Maximum intensity projections of confocal images of isolated pupal macrophages. Cell lineage analysis was done using *tau*>G-TRACE. Green arrowheads mark a GFP-positive lamellocyte derived from PSC cells. Other lamellocytes are GFP negative and may thus be differentiated from other cellular sources. Atilla-positive lamellocytes are marked by yellow arrowheads. Scale bars: 10 µm. (E) Quantification of *tau*>G-TRACE GFP-positive lamellocytes. *n*=157 lamellocytes. (F-G′) Third instar larval lymph gland expressing GFP (green) under the control of *tau*-Gal4 driver co-stained with DAPI (white). (F′,F″) Control lymph gland with prominent expression of GFP in the PSC region of primary lobes. (G′,G″) Wasp-infested lymph gland disintegrates prematurely resulting in a reduced overall cell number. Scale bars: 100 μm (F,G); 50 µm (F′,G′). (H,I) Quantification of *tau*-marked PSC cells (H) and overall lymph gland cell number (I) stained with DAPI confirmed a premature dissociation of the Wasp-infested lymph glands. *P=0.013, **P=0.01 (Mann–Whitney test). Error bars represent s.d. *n*=10 lobes each. (J,K) Maximum intensity projections of confocal images of pupal hemocytes. Phalloidin was used to stain the actin cytoskeleton (F-actin). The PSC cell is marked by EGFP expression driven by *kn*-Gal4 (J) and *tau*-Gal4 (K). Green arrowhead indicates PSC cells. Scale bars: 10 µm. The data show representative images replicated in three independent experiments.

Adult PSC-like cells have recently been shown to proliferate upon bacterial infection (Boulet et al., 2021). To test whether PSC cells further respond to parasitoid wasp infestation, we made use of the G-TRACE (GAL4 technique for real-time and clonal expression) lineage-tracing system (Fig. 6A; Evans et al., 2009). This method uses a cell type-specific Gal4 driver and a UAS-RFP construct to mark Gal4-positive cells with RFP. Gal4 also induces Flp expression, which removes the stop codons in a ubi-FRT-Stop-FRT-GFP construct, resulting in stable GFP expression. Hence, Gal4-positive cells are RFP and GFP positive, whereas offspring cells are GFP positive but RFP negative (Fig. 6A). Activation of G-TRACE with *tau*-Gal4 resulted in labeling of PCS cells (RFP^+^/GFP^+^) of third instar lymph gland. No expression could be observed in other cells outside the PSC (Fig. 6B-B‴). For wasp infestation, female wasps were added into vials for infection treatments and allowed to infect larvae 48 h after egg transfer.

Using *tau*-Gal4 to drive G-TRACE, we observed small RFP^+^/GFP^+^ cells with the PSC-specific spiky morphology (Fig. S7). After wasp infestation, we additionally detected some GFP^+^/RFP^−^ cells with prominent lamellocyte morphology (Fig. 6C-C‴), which were large, flat, Atilla-positive cells with a very prominent dense actin cytoskeleton meshwork (Fig. 6D-D‴). Quantification revealed that approximately 3.2% of all Atilla-positive lamellocytes derived from PSC cells upon wasp infestion, whereas the majority of Atilla-positive lamellocytes cells had different origins (Fig. 6E). We could not detect any additional lymph gland *tau* expression even upon parasitoid wasp infestation. Instead, we observed a progressively reduced number of *tau-*positive cells which corresponds with an earlier lymph gland dispersal induced by wasp infection (Fig. 6F-G′; quantification in Fig. 6H,I). Thus, PSC cells can give rise to lamellocytes in response to parasitism, a cellular plasticity mechanism that might persist to adults. Supporting this notion, we could also isolate single *kn*- and *tau*-positive PSC cells from adult flies with a characteristic spiky morphology reminiscent of those cells observed in pupa (Fig. 6J,K).

### PSC cells are migratory immune-responsive cells

**Fig. 7.**
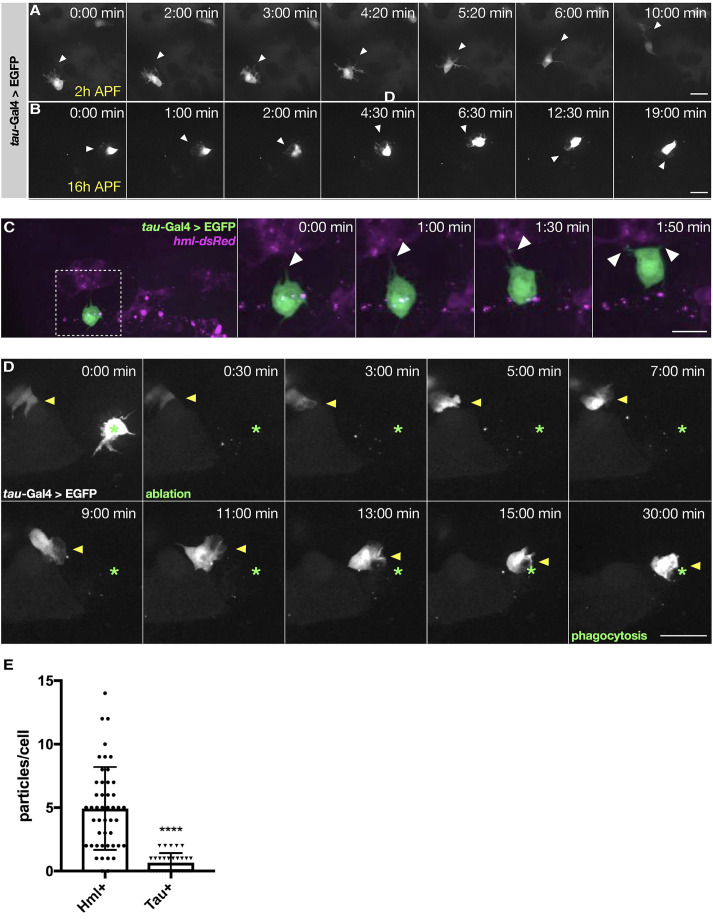
**PSC cells are highly motile and immune-responsive cells.** (A,B) Frames of spinning disk time-lapse movies of randomly migrating 4 h (A) and 16 h (B) APF pupal hemocytes marked by GFP expression under the control of *tau*-Gal4. The time point of each image is annotated. Scale bars: 20 µm. (C) Co-labeling of EGFP-marked PSC cell with *Hml*-dsRed-positive plasmatocytes in a 16 h APF pupa. The PSC cell extends dynamic filopodial protrusions towards plasmatocytes. White arrowheads indicate filopodial protrusions of PSC cells. Dashed box indicates the area enlarged in subsequent images. (D) Still images of a spinning disk time-lapse movie of a *tau*-marked PSC cell (marked by a yellow arrowhead) migrating towards the wound site. At the wound site, the *tau*-marked PSC cell forms a typical phagocytic cup. An ablated PSC cell is marked by an asterisk. (E) Quantification of phagocytosis activity of isolated *tau*-marked cells compared with professional *Hml*-marked phagocytes. ****P<0.0001 (Mann–Whitney Test). Error bars represent s.d. *n*=30 cells. The data show representative images replicated in three independent experiments. Scale bars: 20 μm.

To characterize PSC cells *in vivo*, we used *kn*-Gal4 as well as *tau*-Gal4 drivers to perform live-cell imaging in developing pupae (Movies 1 and 2; Fig. 7). High-resolution live cell imaging microscopy of 4 h APF pupae revealed migrating single *tau*-marked PSC cells that form polarized dynamic lamellipodial and filopodial protrusions and start to redistribute from the dorsal patches of the body wall, as similarly observed for plasmatocytes (Fig. 7A,B; Movie 2; Lehne et al., 2022; Sander et al., 2013). As already found in pupal bleeds, the overall number of cells marked by *tau*-Gal4 in 16 h APF pupae was significantly lower than those marked by the *kn*-Gal4 driver (also compare with Fig. 5K). PSC cells were highly motile but much smaller than *Hml*-marked plasmatocytes (Movie 2). This difference in cell size became more evident later in pupal development (>16 h APF) when cell dispersal in the pupa is further advanced (Movies 1, 2). Compared with *Hml*-marked plasmatocytes, which were more evenly distributed in the whole developing pupa, PSC cells became more restricted to the abdomen during pupal development until hatching. Co-labeling with *Hml*-dsRed-positive plasmatocytes further revealed close contact of single EGFP-marked PSC cells with highly abundant plasmatocytes, interacting by multiple fine filopodial protrusions reminiscent of lymph gland PSC cells (Fig. 7C; Movie 2). Remarkably, *tau*-marked PSC cells were highly motile and immune responsive, as shown by laser-ablation wounding experiments (Fig. 7D, Movie 3). Upon wounding, cells switched from random to directed migration towards the wounding site, as indicated (Fig. 7D, Movie 3). At the wound site, *tau*-marked PSC cells frequently formed typical phagocytic cups to engulf cell debris (Fig. 7D, Movie 3). Quantification of EGFP-marked cells sorted by fluorescence-activated cell sorting confirmed limited phagocytosis activity compared with professional phagocytic *Hml*-marked plasmatocytes (Fig. 7E). Interestingly, in rare cases we observed dividing *tau*-positive cells in live-imaging experiments (Movie 4).

Combined, these data strongly suggest a dual function of PSC cells in *Drosophila* hematopoiesis. Apart from its well-known important function as the larval hematopoietic niche, our data suggest that PSC cells can also function as an immune-responsive cell group capable of blood cell differentiation upon immune challenge later in development.

## DISCUSSION

In this study, we present the first molecular description of pupal *Drosophila* blood cells and provide insights into blood cell functional diversification and plasticity during pupal metamorphosis. Our scRNA-seq data analysis revealed cell populations already described in embryonic or larval stages of hematopoiesis, but also new subgroups with distinct transcriptional signatures appearing with the onset of metamorphosis and persisting into adulthood.

The most abundant *srp* high-expressing undifferentiated PL represents a good example of a cluster present across all stages of hematopoiesis, previously annotated as PL-0 or PLASM in larvae (Cattenoz et al., 2020; Leitão et al., 2020), as PM1/PM2 or PL2 in lymph glands (Cho et al., 2020; Girard et al., 2021) and as Plasmatocytes nAChRalpha3 and trol-high in adult flies (Li et al., 2022). Compared with that, the Osiris-PL cluster likely represents a new cell population of specialized hemocytes specific for metamorphosis, characterized by its prominent expression of Grindelwald (Grnd), one of two TNF receptors in flies. Developmental transitions such as metamorphosis represent a dramatic phase marked by programmed cell death and inflammatory signals induced by increased steroid hormone secretion. Most of the larval tissues are destroyed and hemocytes rapidly upregulate cell motility and phagocytosis of apoptotic debris. In mammals, members of the TNF family play an important role in the regulation of cellular proliferation, differentiation and programmed cell death. Thus, the Osiris-PL cluster might be such inflammatory immune cell cluster important for the regulation of dramatic cellular changes and dynamic tissue remodeling during metamorphosis. The exact role of these cells also remains to be investigated.

The Chitinase-PL and Adhesive-PL clusters also represent cell clusters without significant correlates from other developmental stages. Both effector cell clusters share increased expression of several genes implicated in cell–cell adhesion and septate junction formation required for encapsulation of parasitic wasp eggs. Most known parasitoid wasp species attack the larval or pupal stages of *Drosophila*. Whereas *Trichopria drosophilae* infect the pupal stages of the host, females of the genera *Leptopilina* and *Ganaspis* attack the larval stages (Chen et al., 2018; Small et al., 2012). Core components of the Toll pathway that are highly upregulated in the Adhesive-PL cluster are known to control the immuno-genetic circuit of host immune response against parasitoid wasp attack by activating the NF-κB transcription factor *dorsal*. Future detailed studies are required to dissect whether and how these newly identified plasmatocyte effectors might contribute to this fascinating process of encapsulation.

We also identified a small group of circulating pupal immune cells that resembles the PSC, which acts as a niche to regulate the differentiation of effector types from progenitors in the lymph gland, but also participates in the larval response to wasp parasitization (Banerjee et al., 2019). PSC cells are clearly distinguished by their co-expression of Antp and Knot from other regions within the lymph gland (Crozatier et al., 2004; Lebestky et al., 2000; Mandal et al., 2007). In this study, we confirmed *tau* as an additional specific marker for PSC cells (Cho et al., 2020). Circulating *kn* lineage-traced cells have been also recently identified as small subset of *domeMeso*-positive hemocytes with a characteristic fusiform shape in the adult hemolymph that derived from the lymph gland PSC and account for 3-4% of hemocytes in the adult (Boulet et al., 2021). Recent scRNA-seq analyses suggested that PSC-like cells expressing *kn* and *Antp* are also present in larval hemolymph and have been interpreted as a rare subclass of plasmatocytes that account for <1% of circulating hemocytes (Cattenoz et al., 2020, 2021; Tattikota et al., 2020). Larval PSC-like cells probably correspond to the group of so-called primocytes that has been recently described by Fu and colleagues at the same time (Fu et al., 2020). Primocytes also account for <1% of circulating larval hemocytes and express characteristic marker genes, including *srp*, *Antp* and *kn*, but not hemocyte markers such as *He*, *Pxn* and *crq* (Fu et al., 2020). In this study, we confirmed the presence of such a subclass of circulating *kn*-positive, PSC-like cells in the larval and pupal hemolymph. However, *kn*-positive cells represent at least two morphologically distinct cell types: plasmatocyte-shaped cells and small spiky cells with several filopodia-like protrusions, as described for fusiform cells in adults (Boulet et al., 2021). In contrast to *kn*, the PSC marker *tau* exclusively labeled small spiky cells only present in the pupa. In larva, we also found a small subset of *kn*-positive cells in larval bleeds, but all labeled larval cells exhibited a plasmatocyte-like round morphology, suggesting that these morphologically different cells have distinct origins. Thus, *kn*-positive cells might represent PSC cells as well as other immune cell populations derived from the lymph gland posterior lobes, as previously suggested (Benmimoun et al., 2012; Ghosh et al., 2015; Rodrigues et al., 2021). Using *tau* as a specific marker for PSC cells, we could visualize for the first time this new hemocyte class *in vivo* by high-resolution, live-imaging microscopy. PSC cells are highly motile and immune-responsive cells. Co-labeling experiments further revealed close contact of rare PSC cells with highly abundant plasmatocytes, interacting by multiple filopodial protrusions, reminiscent of lymph gland PSC cells (Krzemień et al., 2007; Mandal et al., 2007). Thus, motile PSC cells might control peripheral plasmatocytes, like the cells of the posterior signaling center controlling hemocyte maturation in the primary lobe of the lymph gland. Our data further suggest that that PSC cells can transdifferentiate to lamellocytes in response to parasitoid wasp infection. A recent study also reveals an important function of the PSC cells is to trigger plasmatocytes upon bacterial infection (Boulet et al., 2021). Thus, our results corroborate recent findings that PSC cells might also serve as a plastic blood cell type upon immune challenges and show that this function is not limited to the adult stage (Boulet et al., 2021). The contribution of PSC-derived lamellocytes or plasmatocytes upon immune challenge, however, remains to be further investigated.

## MATERIALS AND METHODS

### *Drosophila* genetics

Fly husbandry and crossing were carried out according to standard methods. Crosses were maintained at 25°C. The following fly lines were used: *Hml*-DsRed (D. Siekhaus, Institute of Science and Technology, Austria), srpHemo-3xmCherry (Gyoergy et al., 2018); *srp*-GFP (Vienna Drosophila Resource Center, 318053); *Hml*Δ-Gal4, UAS-eGFP (Sinenko and Mathey-Prevot, 2004); UAS-eGFP (BL 6874). *tep4*-Gal4 (BL 76750), Ance^MiMiC^-GFP (BL 59828), kn-Gal4 (BL 67516), Antp-Gal4 (BL 26817), ham(GMR80G10)-Gal4 (BL 40090), *tau*-Gal4 (BL 77641), CG31174^MiMic^-GFP (BL 24040); UAS-mCherry-NLS (BL 38425). G-TRACE (UAS-RedStinger, UAS-FLP, Ubi-p63E(FRT. STOP)Stinger) (BL 28281).

For wasp infestation, 10-15 female and three to five male flies were transferred to a fresh food vial and placed at 25°C for 48 h. Flies were then removed and five to eight females and three to five male parasitoid wasps of the species *Leptopilina boulardi*, strain G401, were added to parasitize the fly larvae at 25°C. After 3 days, wasps were removed before dissecting pupae as described below. Before dissection, parasitization was confirmed by the presence of encapsulated eggs or wasp larvae visible through the pupal case.

### Isolation of *Drosophila* hemocytes for scRNA-seq

Fifty to sixty 2-10 h APF pupae were collected and washed in 1× PBS. The pupae were then transferred to 1× Schneider's *Drosophila* medium (Gibco) supplemented with 10% fetal bovine serum, 50 units/ml penicillin and 50 µg/ml streptomycin and opened using forks to rinse out the hemolymph. Cells were filtered through a 50-µm cell strainer and collected in a total volume of 500 µl. Subsequently, centrifugation at 500 ***g*** (at 4°C for 20 min) was performed. The supernatant was discarded, and the pellet was resuspended with 500 µl PBS+0.01% bovine serum albumin for single-cell sequencing of live cells. For fixed samples, the previous pellet was resuspended with 100 µl 1× PBS and subsequently fixed by adding 4 volumes of ice-cold 100% methanol (final concentration of 80% methanol in PBS) and thoroughly mixed with a pipette. Cells were stored at −20°C until use (1-3 days). For single-cell sequencing, cells were moved to 4°C and kept on ice throughout the procedure. Fixed cells were pelleted at 500 ***g*** for 15 min and rehydrated in 500 µl PBS+0.01% bovine serum albumin. For datasets 1 and 2 we used fixed samples, whereas we used live cells for dataset 3. All three replicates were used for subsequent cluster analyses.

### scRNA-seq of *Drosophila* pupal hemocytes

The Takara ICELL8 5184 nano-well chip was used with the full-length SMART-Seq ICELL8 Reagent Kit. Cell suspensions were fluorescently labeled with live/dead stain, Hoechst 34580 (Thermo Fisher Scientific) and propidium iodide (NucBlue Cell Stain Reagent, Thermo Fisher Scientific) for 15 min prior to being dispensed into the Takara ICELL8 5184 nano-well chip. CellSelect Software (Takara Bio) was used to visualize and select wells containing single and live cells. Next, cDNA was synthesized via oligo-dT priming in a one-step RT-PCR reaction. P5 indexing primers for subsequent library preparation were dispensed into all wells receiving a different index, in addition to Terra polymerase and reaction buffer. Transposase enzyme and reaction buffer (Tn5 mixture) were dispensed to selected wells. P7 indexing primers were dispensed to wells. Final Illumina libraries were amplified and pooled as they were extracted from the chip. Pooled libraries were purified and size selected using Agencourt AMPure XP magnetic beads (Beckman Coulter) to obtain an average library size of 500 bp. A typical yield for a library comprising ∼1300 cells was ∼15 nM. Libraries were sequenced on the HiSeq 4000 (Illumina) to obtain on average ∼0.3 Mio reads per cell (SE; 50 bp). A list of Gal4-enhancer trap and GFP-exon trap fly lines used for *ex vivo* validation can be found in Table S19.

### Bioinformatic analysis

Raw sequencing files (bcl) were converted into a single fastq file using Illumina bcl2fastq software (v2.20.0.422) for each method. Each fastq file was de-multiplexed and analyzed using Cogent NGS analysis pipeline (CogentAP) from Takara Bio (v1.0). In brief, the ‘cogent demux’ wrapper function was used to allocate the reads to the cells based on the cell barcodes provided in the well-list files. Subsequently, the ‘cogent analyze’ wrapper function performed a preliminary analysis, including: read trimming with cutadapt (version 3.2); genome alignment to *Drosophila melanogaster* Version 103 using STAR (version 2.7.2a); read counting for exonic, genomic and mitochondrial regions in *Drosophila melanogaster* genes from ENSEMBL gene annotation version 103 using featureCounts (version 2.0.1); and summarizing the gene counts into gene matrices with number of reads expressed for each cell in each gene. Raw gene matrices underwent quality-control filtering for cells and genes using the following parameters: for cells, only those with at least 10,000 reads associated to at least 300 different genes were kept, and, for genes, only those containing at least 100 reads mapped to them from at least three different cells were kept. Subsequent analyses were performed using Seurat v4.1.1 (Stuart et al., 2019) in Rstudio 2022.02.2. Low-quality cells were filtered out based on the number of detected genes and the percentage of mitochondrial genes (Fig. S1A). Batch correction between the three replicates was performed, UMAP coordinates were calculated and unsupervised clustering was performed with 16 dimensions and the default resolution factor. Clustering was controlled by testing for sufficient distinct marker genes using the ‘FindAllMarkers()’ command and two clusters were merged due to insufficient distinct markers (resulting in Undifferentiated-PL-1) (Script 1; https://github.com/KatjaRM/scRNAseq-pupal-hemocyte/blob/main/Script_1.R). Pseudotime analysis was performed using monocle3 v1.0.0 using UMAP coordinates calculated with Seurat v4.1.1 and with learn_graph_control=list(ncenter=480) in the ‘learn_graph()’ command (Script 2; https://github.com/KatjaRM/scRNAseq-pupal-hemocyte/blob/main/Script_2.R). SCENIC analysis was performed with SCENIC v1.3.1 using the cisTarget v8 motif collection mc8nr (Script 3; https://github.com/KatjaRM/scRNAseq-pupal-hemocyte/blob/main/Script_3.R). For a GO term analysis we used dDEGs identified using ‘FindMarkers()’. Gene names were converted to Flybase IDs and analyzed with DAVID 2021. Cross-dataset analysis was performed using Seurat v4.1.1, including batch correction and UMAP calculation (Script 4; https://github.com/KatjaRM/scRNAseq-pupal-hemocyte/blob/main/Script_4.R). Scales in Seurat expression plots and maps display the expression in log[(UMI+1/total UMI)×10^4^]. To determine M-phase gene expression, we monitored the expression of the M-phase-specific genes *polo*, *aurB* and *Det* as applied by Cho et al. (2020).

### Immunohistochemistry

Pupal macrophages were isolated as described previously (Lehne et al., 2022). In short, white to light brownish prepupae (2-10 h APF) were collected and washed in 1× PBS. The prepupae were then transferred to 1× Schneider's *Drosophila* medium (Gibco) supplemented with 10% fetal bovine serum, 50 units/ml penicillin and 50 µg/ml streptomycin and opened using forks to rinse out the hemolymph. Cells were spread on glass coverslips, previously coated for 30 min with ConcanavalinA (0.5 mg/ml, Sigma-Aldrich), for 1 h at 25°C. The supernatant was removed and adherent cells were subsequently fixed for 15 min with 4% paraformaldehyde in 1× PBS at room temperature (RT). Cells were rinsed with 1× PBS+0.1% Triton X-100 followed by three washing steps with 1× PBS. If no antibody staining was performed, the treatment with the PBS-Triton solution was omitted. Cells were stained with primary antibody for 2 h at RT and secondary antibody with Phalloidin and DAPI for 1 h at RT in a humidified dark chamber. Stained cells were mounted in Mowiol 4-88 (Carl Roth). The following primary antibodies were used: anti-Atilla (1:10; Kurucz et al., 2007), anti-Hnt (1:5, 1G9 from Developmental Studies Hybridoma Bank), anti-Grnd (1:500; Andersen, et al., 2015), anti-Tpl94D (1:200; Kimura and Loppin, 2016). The following secondary antibody was used: polyclonal Alexa Fluor-647-conjugated goat-anti-mouse (1:1000; A21236, Invitrogen). F-actin staining was visualized using Phalloidin-Fluor 405 (1:100; 176752, Abcam) or Alexa Fluor-Phalloidin 568 (1:100; A12380, Invitrogen) and nuclei by DAPI staining (1 µg/ml; 62248, Thermo Fisher Scientific).

### Cell sorting

For cell sorting, pupal hemocytes were obtained as for immunostainings and filtered through a 50 µm cell strainer. A Bio-Rad S3e cell sorter with ProSort software version 1.6 was used for sorting. Debris was excluded based on FSC/SSC ratio and GFP fluorescence excited with a 488 nm blue laser. Cells were then sorted by high signal at the FL1 detector equipped with a 525/30 excitation filter. w1118 and Hml-Gal4, UAS-eGFP pupal hemocytes were used as negative and positive control, respectively.

### Phagocytosis assay

Pupal macrophages were isolated in 1× Schneider's *Drosophila* medium (Gibco) supplemented with 1 nM phenyl thiourea. After adhering to a glass coverslip for 1 h at 25°C, the medium was gently removed, and replaced with 200 µl of fresh medium supplemented with 1 nM phenyl thiourea and Texas Red-conjugated *Escherichia coli* K-12 particles (Molecular Probes BioParticles^®^ E-2863) for a density of 53,000 particles/mm2 and incubated for 1 h at room temperature in the dark. Cells were then fixed by incubation in 4% paraformaldehyde solution for 15 min at RT. The number of internalized particles per cell was counted for 30 cells per group from confocal images taken on a Leica TCS SP8 with an HC PL APO CS2 63×/1.4 oil objective and Leica Application Suite X (LasX, Version 3.5.2.18963). The results were statistically analyzed using GraphPad Prism version 7 (GraphPad Software).

### Preparation of larval lymph glands

Wandering L3 larvae were anesthetized on ice and placed ventral side up on a silicone dissection pad in a drop of PBS. The larvae were pinned in place with one insect pin at the mouth hooks and one between the posterior spiracles, then sliced open lengthwise with a fine cataract scissor. The ventral cuticle was pinned to the side and the gut and fat body were carefully removed. The larva was fixed by removing the PBS and adding 10 µl of 4% paraformaldehyde for 20 min at RT. The lymph gland was then carefully pulled from the fillet along with the attached brain and transferred to a drop of Fluoromount with DAPI (Invitrogen) on a microscope slide. The brain was severed and removed from the lymph gland before placement of the coverslip. Lymph glands were imaged using a Leica TCS SP8 confocal microscope with a HC PL APO CS2 20×/0.75 air objective.

### Image acquisition and microscopy

Pupae were imaged whole with a Leica Fluorescence Stereo Microscope and Leica Application Suite X (LasX, Version 3.5.2.18963).

Confocal fluorescent images were taken with a Leica TCS SP8 with an HC PL APO CS2 63×/1.4 oil objective and Leica Application Suite X (LasX, Version 3.5.2.18963). Live imaging of macrophage cultures was performed using a Zeiss CellObserver Z.1 with a Yokogawa CSU-X1 spinning disk scanning unit and an Axiocam MRm CCD camera (6.45 µm×6.45 µm) and ZenBlue 2.5 software. Ablation experiments were performed using the UV ablation system DL-355/14 from Rapp OptoElectronics, as reported previously (Lehne et al., 2022).

### Live-cell imaging of pupal hemocytes

Live imaging of pupal macrophages at 4 h APF for random migration as well as at 16 h APF for directed migration experiments were performed as described previously (Lehne et al., 2022). Prepupae were collected at 4 h APF and glued to a glass coverslip on their dorsal-lateral side. Spinning disk time-lapse movies were generated by taking images every 20 s for 30 min. APF pupae (16-18 h) were dissected out of their cuticles and laid on a glass coverslip on their dorsal abdomen. Single cells were ablated as described in the previous section. Spinning disk time-lapse movies were generated by taking images every 30 s for 60 min.

## Supplementary Material

10.1242/develop.201767_sup1 Supplementary information
