# Supplementary material for: Single-cell transcriptomics identifies new blood cell populations in Drosophila released at the onset of metamorphosis
Source: Development. 2023 Sep 27;150(18):dev201767. doi: 10.1242/dev.201767 (PMC10560556; doi:10.1242/dev.201767)
Supplement: Supplementary information [file develop-150-201767-s1.pdf]

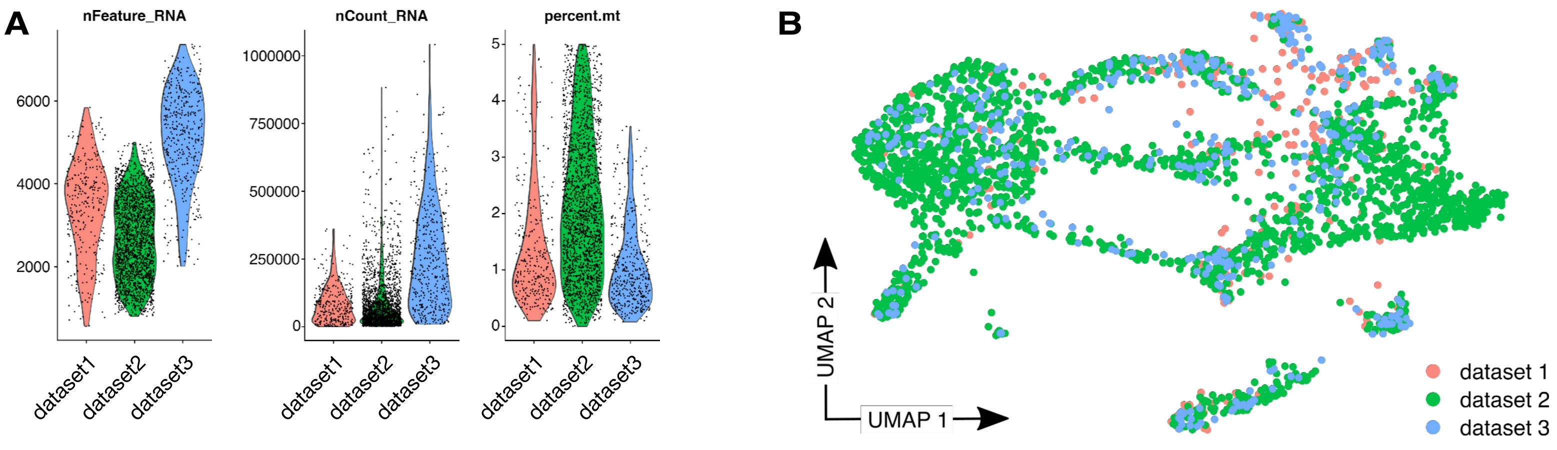

C

	dataset1 (fixed cells)	dataset2 (fixed cells)	dataset3 (live cells)	total
all cells	305	2084	422	**2811**
Undifferentiated PL	23	704	90	**817**
Transitory PL-1	2	81	20	**103**
Transitory PL-2	37	113	51	**201**
Osiris-PL	13	125	28	**166**
Spermatid-marker-PL	29	182	32	**243**
Lsp-Bomanin-PL	93	194	78	**365**
OxPhos-PL	4	328	4	**336**
Chitinase-PL	12	8	14	**34**
Adhesive-PL	10	75	13	**98**
AMP-PL	24	11	22	**57**
Secretory-PL	10	107	14	**131**
PSC	1	12	3	**16**
Neuron	37	103	32	**172**
Muscle	10	41	21	**72**

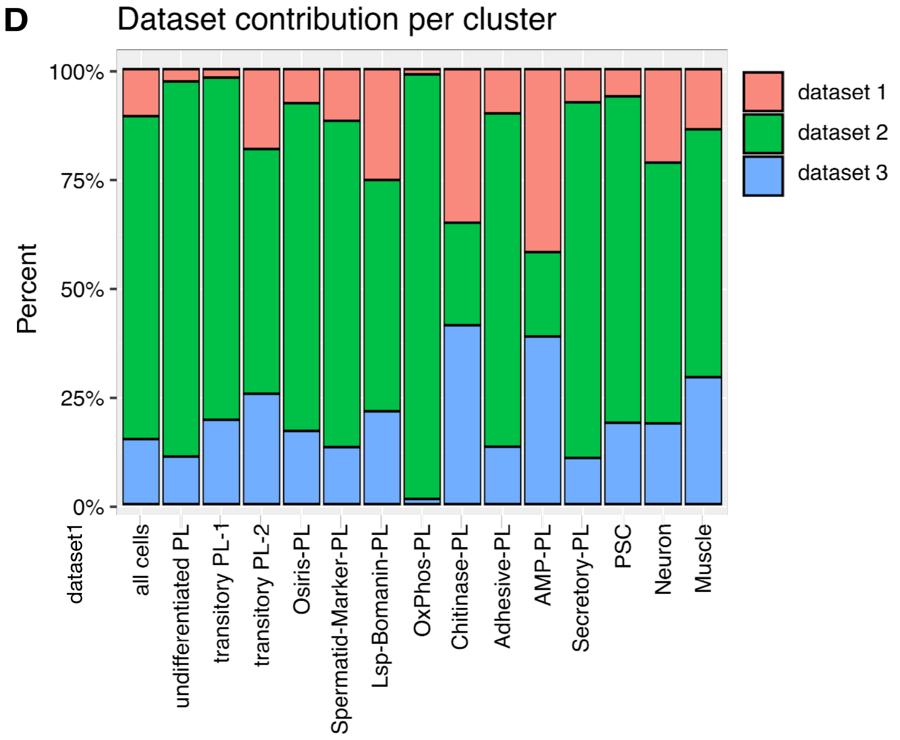

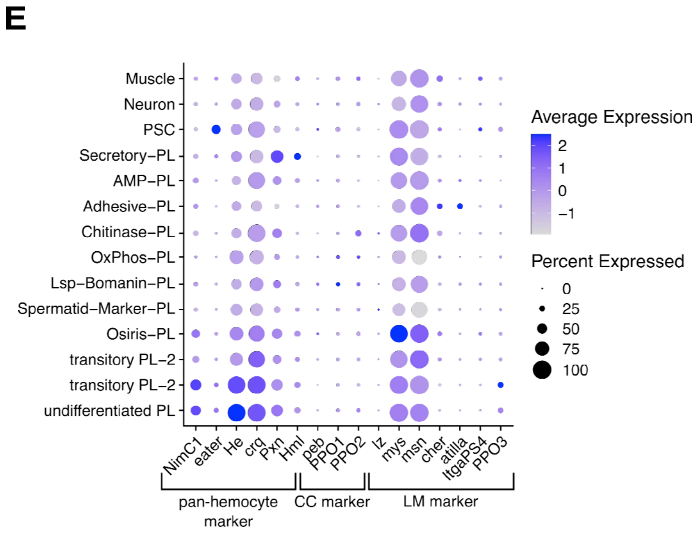

Fig. S1. scRNA-seq dataset quality

A) Violin plots representing the number of Features, UMI counts and percent of mitochondrial genes per cell and per dataset. B) UMAP plot with cells labeled by dataset origin. C, D) Contribution of cells per dataset in a table (C) or represented as a barplot (D). Note that all datasets (two fixed and one live cell dataset) contribute to every cluster. E) Average expression and percent expression per cluster of known pan-hemocyte, crystal cell and lamellocyte markers.

Development • Supplementary information

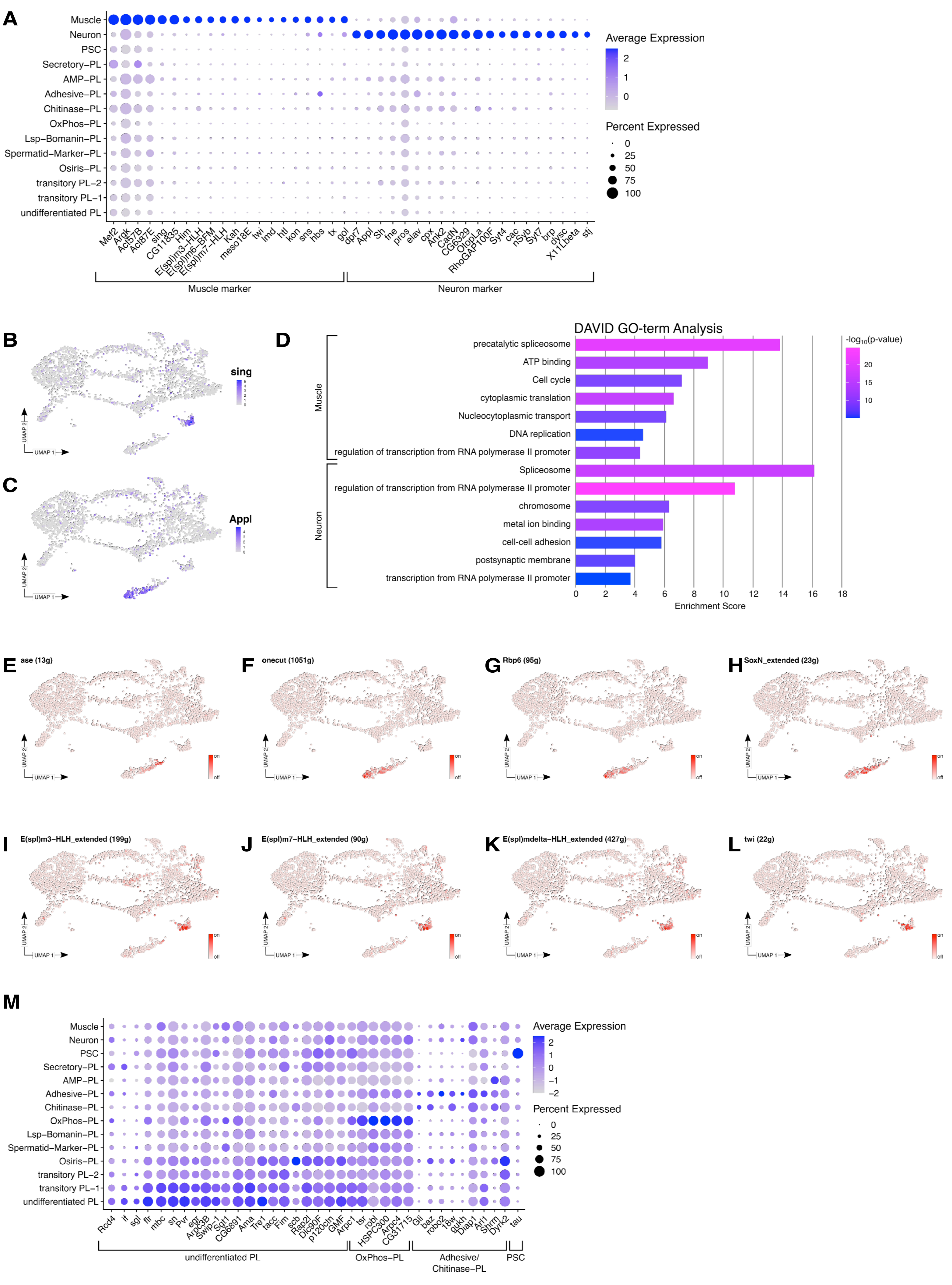

Fig. S2. Annotation of muscle and neuron clusters

A) Dotplot showing the average expression and percent expression of muscle and neuron markers. B-C) Expression of the muscle marker *sing* (B) and the neuron marker *Appl* (C) on the UMAP plot. D) DAVID 2021 GO-term analysis of Neuron and Muscle markers. For each of the top seven annotation cluster a representative term is presented. Barplot shows enrichment score of the annotation cluster and p-value of the GO-term. E-L) SCENIC determined activity of transcription factors active in Neuron (E-H) or Muscle (I-L) on the UMAP plot. E) *ase*, F) *onecut*, G) *Rbp6*, H) *SoxN*, I) *E(spl)m3-HLH*, J) *E(spl)m7-HLH*, K) *E(spl)mdelta-HLH*, L) *twi*. M) Average expression and percent expression per cluster of cytoskeleton and cell motility genes identified in a bulk RNA-sequencing experiment comparing larval and pupal hemocytes.

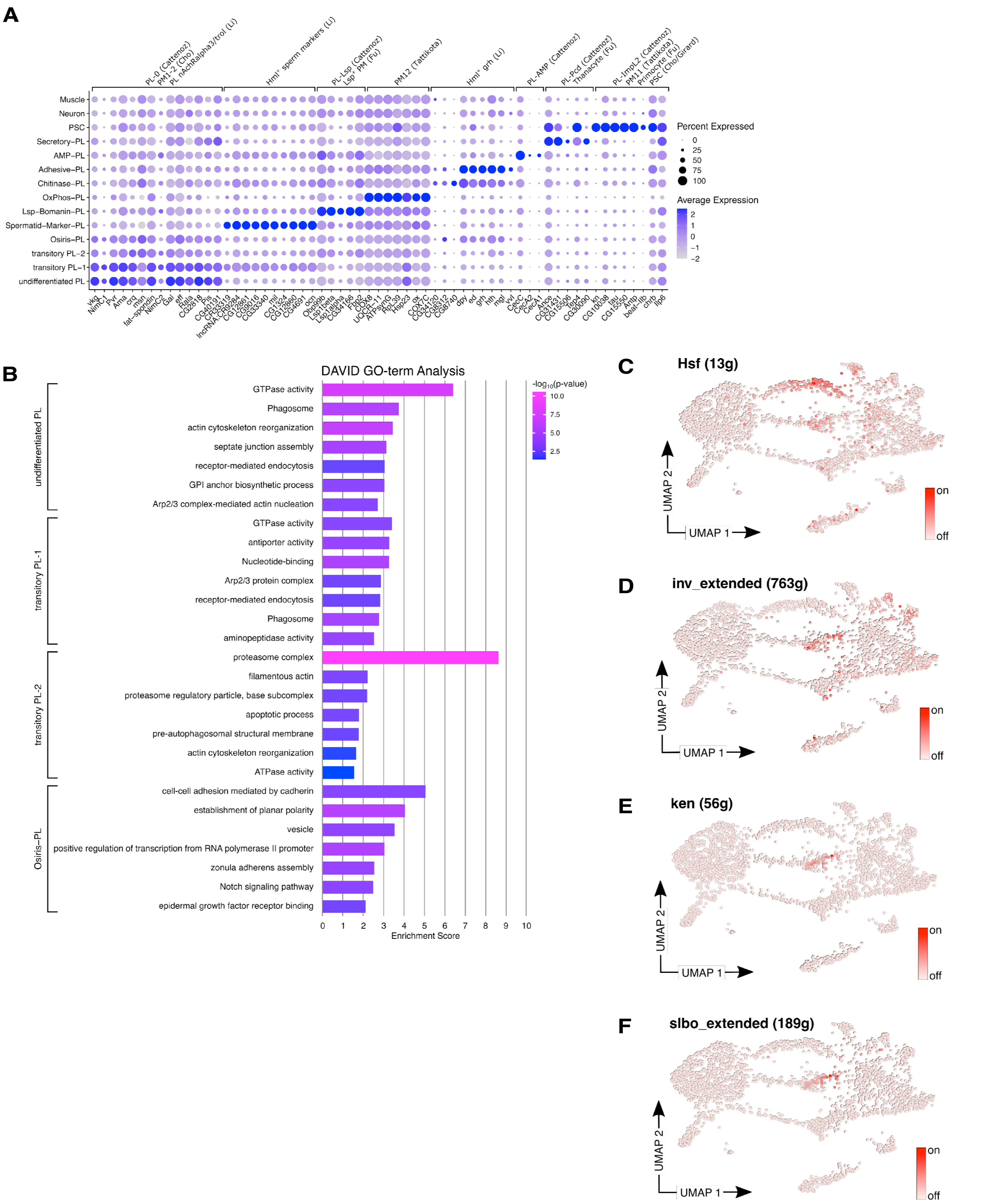

Fig. S3. Dataset correlation and annotation of undifferentiated hemocyte clusters

A) Dotplot of the average and percent expression of hemocyte subtype specific markers identified in other studies (Cattenoz et al., 2020, Cho et al., ,2020, Li et al., 2022, Fu et al., 2020, Tattikota et al., 2020, Girard et al., 2021). This table only includes markers that display cluster specific expression in our dataset. B) Marker genes of undifferentiated PL, transitory PL-1-2 or Osiris-PL were analyzed with DAVID 2021. For the top seven annotation cluster one representative GO-term is shown. Barplot shows enrichment score of the annotation cluster and is colored by GO-term p-value. C-F) Activity of transcription factors on the UMAP plot as identified by SCENIC. C) Hsf, D) inv, E) ken, F) slbo.

Development • Supplementary information

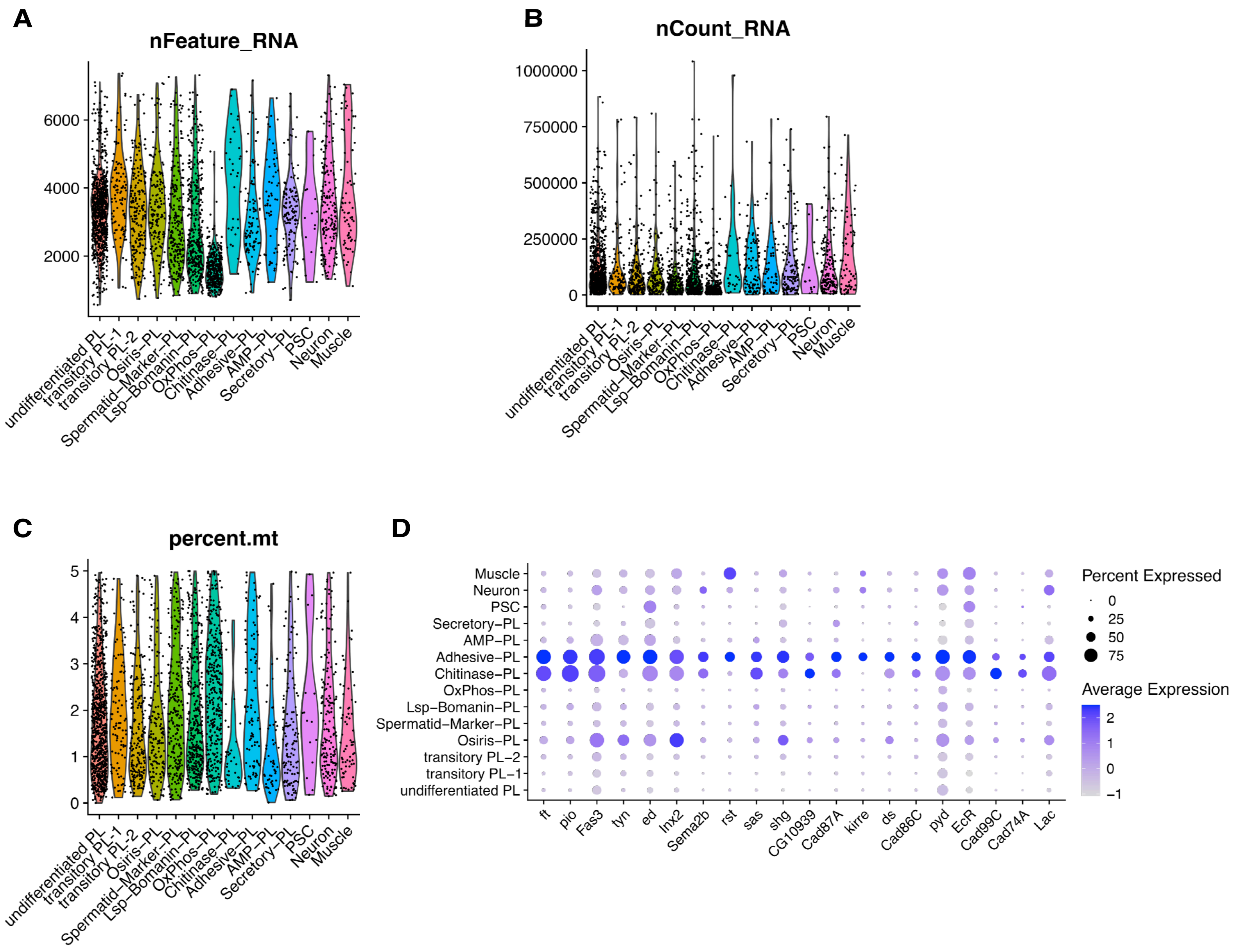

Fig. S4. Adhesive and Chitinase-PL express genes important for cell adhesion

A-C) Violin plot showing the number of (A) features, (B) UMIs or (C) percent of mitochondrial genes per cluster. D) Average and percent expression of genes implicated in cell adhesion identified as markers for Adhesive-PL. Note that most genes are also expressed in Chitinase-PL.

Development • Supplementary information

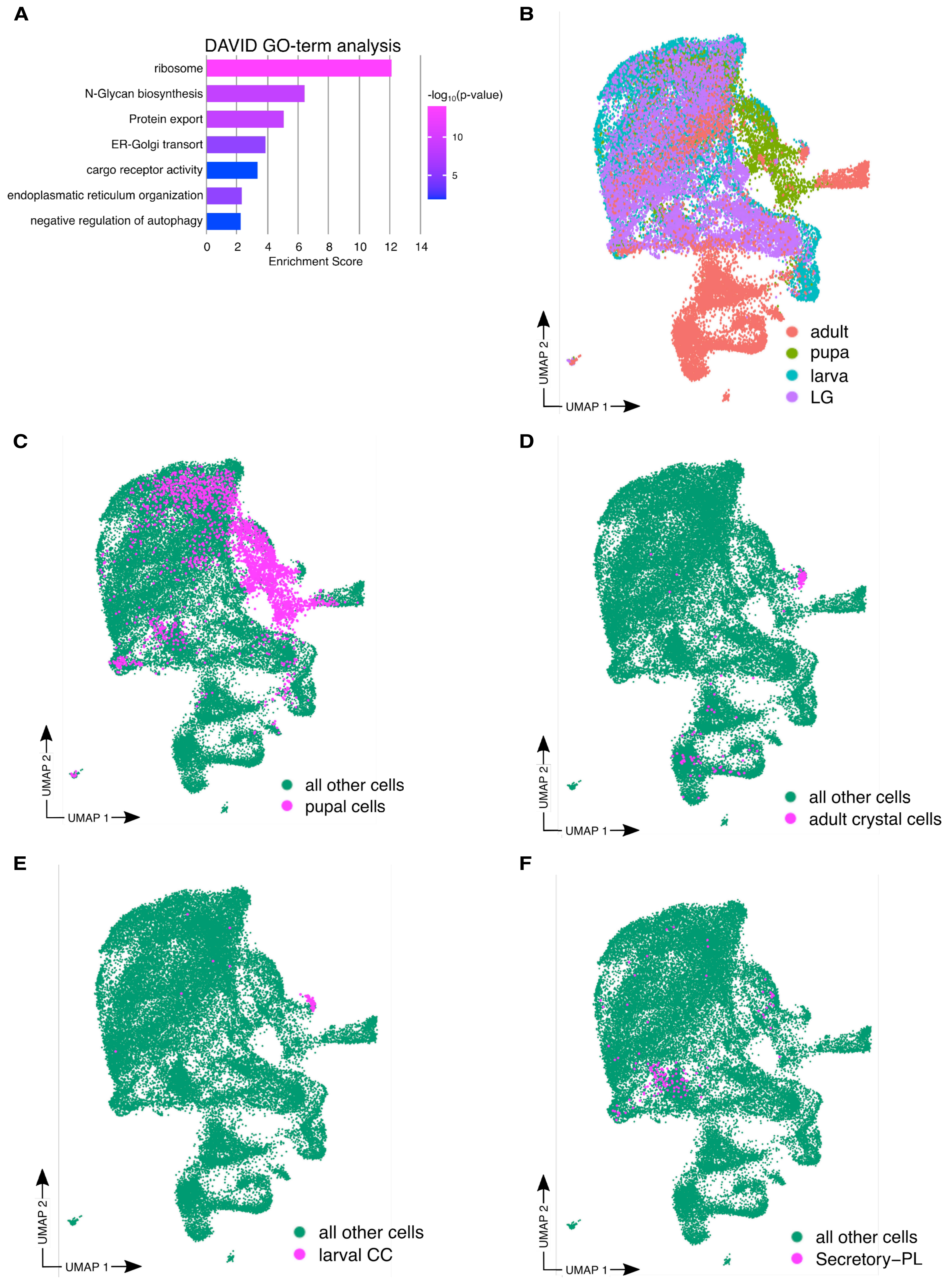

Fig. S5. Secretory-PL are distinct from crystal cells.

A) GO-term analysis of Secretory-PL markers performed with DAVID 2021. Representative GO-terms for the top seven annotation clusters are shown. Enrichment score of annotation clusters and p-value of GO-terms are presented in A barplot. B-F) UMAP plots of integrated hemocyte datasets across different developmental stages (this study; Cho et al., 2020; Cattenoz et al., 2020; Li et al., 2022). B) Labelled by dataset. C) Pupal cells are highlighted in magenta. D) Adult crystal cells are highlighted in magenta. E) Larval crystal cells are shown in magenta. F) Pupal Secretory-PL are represented in magenta.

Development: doi:10.1242/dev.201767: Supplementary information

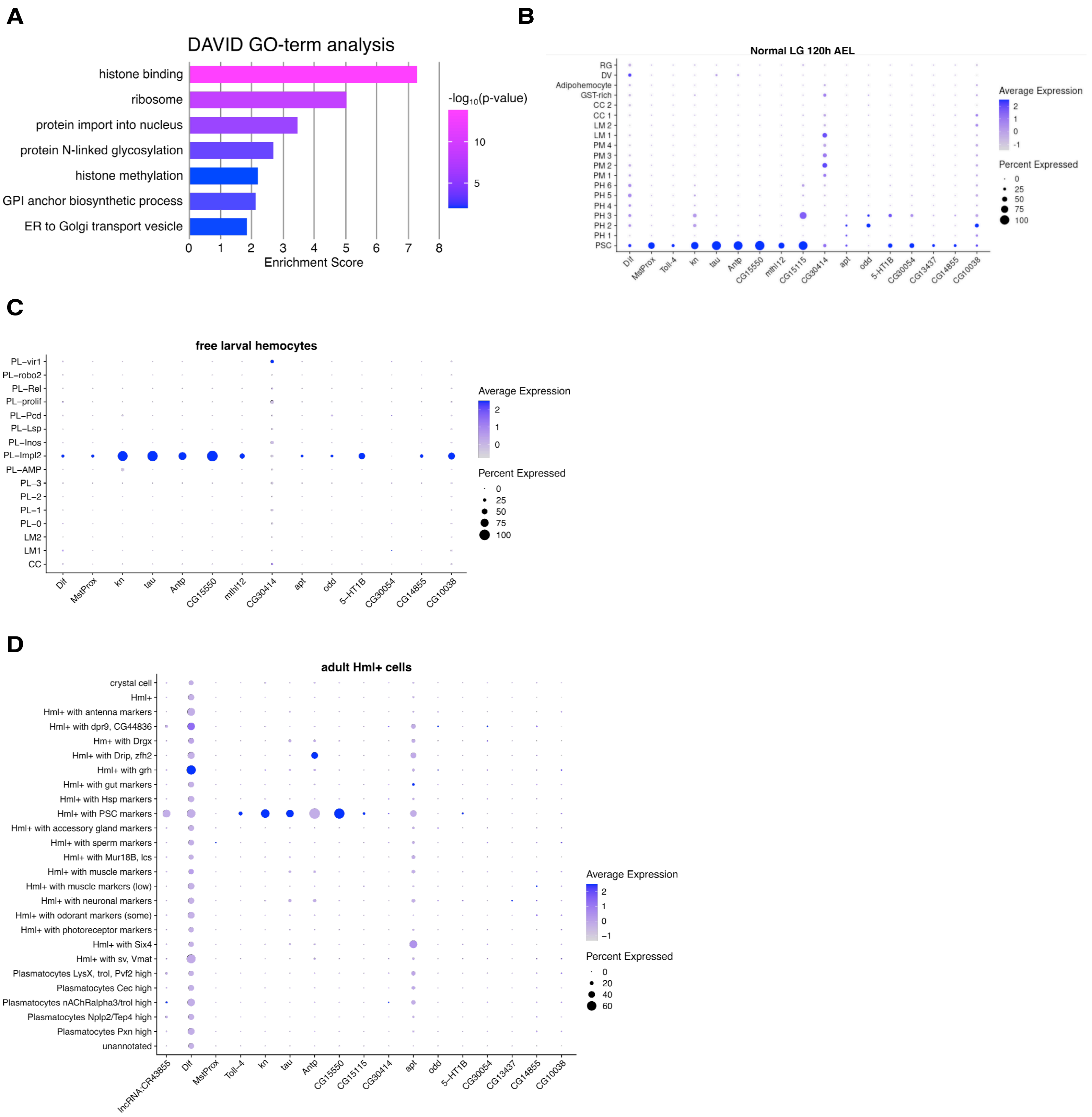

Fig. S6. Identification of PSC-like clusters in other developmental stages.

A) PSC markers were analysed with DAVID 2021 and representative GO-terms of the top seven GO-terms are presented. Barplot shows the enrichment score of the annotation cluster and is colored by p-value of the GO-term. B-D) Dotplots of markers of pupal PSCs showing average expression and percent expression per cluster in B) the normal lymph gland at 120h after egg laying (Cho et al., 2020), C) free larval hemocytes of embryonic origin (Cattenoz et al., 2020) and D) in adult Hml positive cells (Li et al., 2022). D) UMAP plot of integrated datasets across developmental stages showing the location of pupal PSC.

Development • Supplementary information

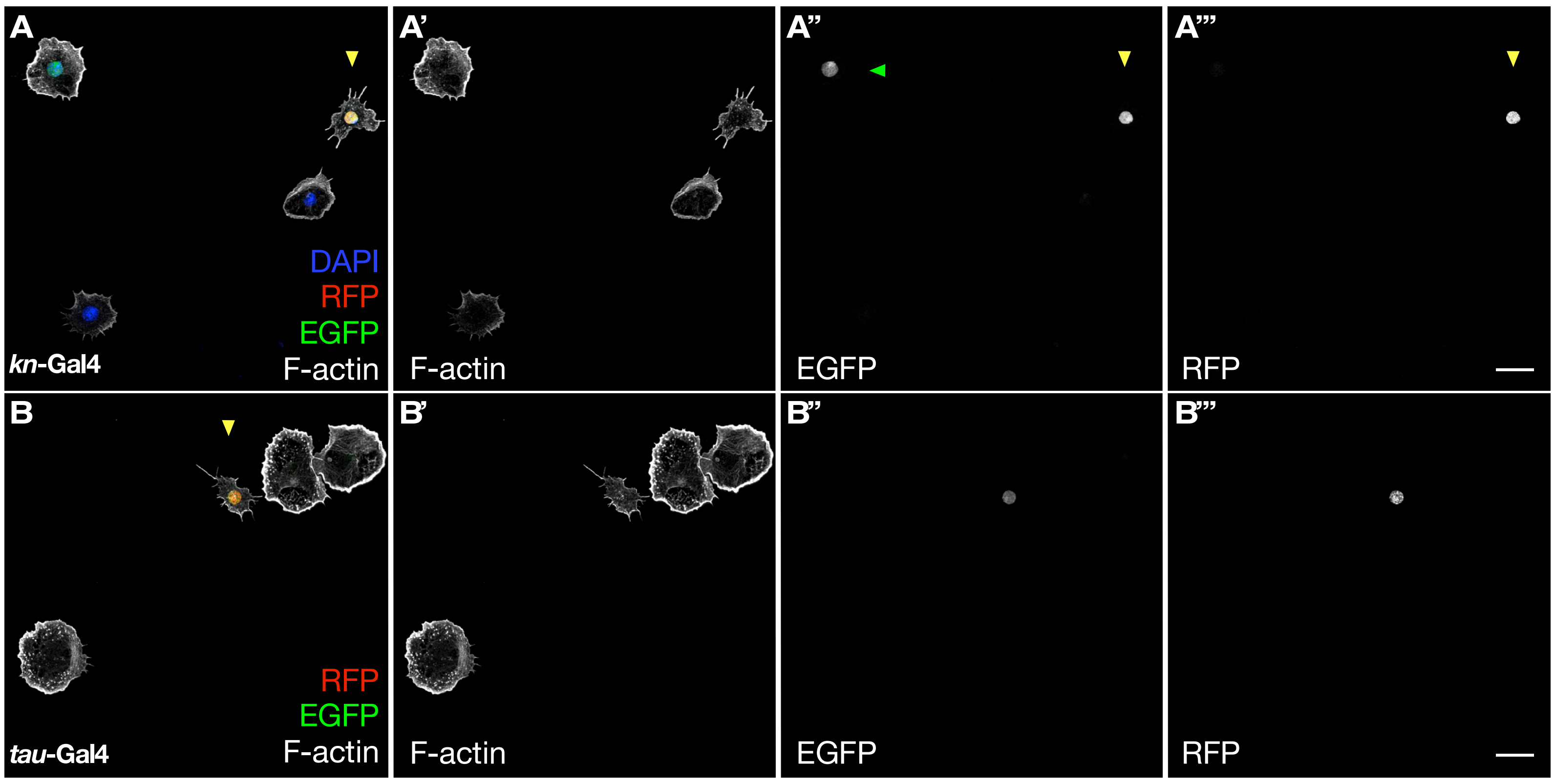

Fig. S7. *tau* is suitable for lineage tracing of PSCs.

(A, B) Cell lineage analysis of non-infested pupal hemocytes using (A) *kn* > G-TRACE. (H) *tau* > G-TRACE. Scale bar represents 10 μm.

Table S1. Pupal blood cell types and likely corresponding cell types identified in studies characterizing other stages of hematopoiesis. Corresponding clusters were identified by examining the respective cluster specific markers in pupal blood cell clusters (compare to Supplementary figure S3A). In addition, we considered previously identified overlap between clusters (Hultmark and Andó, 2022).

Cluster	Equivalent clusters (larva)	Equivalent clusters (primary lobe LG)	Equivalent clusters (adult)
undifferentiated PL	PL-0 (Cattenoz)<br>PLASM1 (Leitão)	PM1-2 (Cho)<br>PL2 (Girard)	Plasmatocytes<br>nAChRalpha3/trol (Li)
transitory PL-1			
transitory PL-2			
Osiris-PL			
Spermatid marker PL			Hml+ with sperm markers (Li)
Lsp Bomanin PL	PL-Lsp (Cattenoz)<br>Lsp+ (Fu)		
OxPhos-PL		PM12 (Cho)	
Chitinase PL			Hml+ with grh (Li)
Adhesive PL			Hml+ with grh (Li)
AMP PL	PL-AMP (Cattenoz)<br>PM7 (Tattikota)<br>AMP (Leitão)	PH4 (Cho)<br>MZ (Girard)	Plasmatocytes Cec and other immunity genes high (Li)
Secretory PL	PL-Pcd (Cattenoz)<br>Thanacyte (Fu)	PP (Girard)	
PSC	PL-ImpL2 (Cattenoz)<br>PM11 (Tattikota)<br>Primocyte (Fu)	PSC (Cho)<br>PSC (Girard)	Hml+ with LG PSC markers (Li)

References:

Cattenoz, P.B., Sakr, R., Pavlidaki, A., Delaporte, C., Riba, A., Molina, N., Hariharan, N., Mukherjee, T., and Giangrande, A. (2020). Temporal specificity and heterogeneity of Drosophila immune cells. EMBO J *39*, e104486.

Cho, B., Yoon, S.H., Lee, D., Koranteng, F., Tattikota, S.G., Cha, N., Shin, M., Do, H., Hu, Y., Oh, S.Y., *et al.* (2020). Single-cell transcriptome maps of myeloid blood cell lineages in Drosophila. Nat Commun *11*, 4483.

Fu, Y., Huang, X., Zhang, P., van de Leemput, J., and Han, Z. (2020). Single-cell RNA sequencing identifies novel cell types in Drosophila blood. J Genet Genomics *47*, 175-186.

Table S1. List of differentially expressed genes across all 14 clusters identified with Seurat v4.1.1 FindAllMarkers() command.

Click here to download Table S1

Table S2. Data associated with Figure 1C. This list contains the top50 genes identified with Seurat v4.1.1 FindAllMarkers() and is an abbreviated version of Table S1.

Click here to download Table S2

Table S3. Differentially expressed genes of undifferentiated PL identified with Seurat v.1.1 FindMarkers() used for DAVID 2021 GO-term analysis.

Click here to download Table S3

Table S4. Differentially expressed genes of transitory PL-1 identified with Seurat v.1.1 FindMarkers() used for DAVID 2021 GO-term analysis.

Click here to download Table S4

Table S5. Differentially expressed genes of transitory PL-2 identified with Seurat v.1.1 FindMarkers() used for DAVID 2021 GO-term analysis.

Click here to download Table S5

Table S6. Differentially expressed genes of Osiris-PL identified with Seurat v.1.1 FindMarkers() used for DAVID 2021 GO-term analysis.

Click here to download Table S6

Development • Supplementary information

Table S7. **Differentially expressed genes of Spermatid-Marker-PL identified with Seurat v.1.1 FindMarkers() used for DAVID 2021 GO-term analysis.**

Click here to download Table S7

Table S8. **Differentially expressed genes of Lsp-Bomanin-PL identified with Seurat v.1.1 FindMarkers() used for DAVID 2021 GO-term analysis.**

Click here to download Table S8

Table S9. Differentially expressed genes of OxPhos-PL identified with Seurat v.1.1 FindMarkers() used for DAVID 2021 GO-term analysis.

Click here to download Table S9

Table S10. Differentially expressed genes of Chitinase-PL identified with Seurat v.1.1 FindMarkers() used for DAVID 2021 GO-term analysis.

Click here to download Table S10

Table S11. **Differentially expressed genes of Adhesive-PL identified with Seurat v.1.1 FindMarkers() used for DAVID 2021 GO-term analysis.**

Click here to download Table S11

Table S12. **Differentially expressed genes of AMP-PL identified with Seurat v.1.1 FindMarkers() used for DAVID 2021 GO-term analysis.**

Click here to download Table S12

Table S13. Differentially expressed genes of Secretory-PL identified with Seurat v.1.1 FindMarkers() used for DAVID 2021 GO-term analysis.

Click here to download Table S13

Table S14. Differentially expressed genes of PSC identified with Seurat v.1.1 FindMarkers() used for DAVID 2021 GO-term analysis.

Click here to download Table S14

Table S15. Differentially expressed genes of Neuron identified with Seurat v.1.1 FindMarkers() used for DAVID 2021 GO-term analysis.

Click here to download Table S15

Table S16. Differentially expressed genes of Muscle identified with Seurat v.1.1 FindMarkers() used for DAVID 2021 GO-term analysis.

Click here to download Table S16

Table S17. Scaled regulon activity identified by SCENIC v1.3.1. A subset of this data is visually presented in Figure 1D.

Click here to download Table S17

Table S18. Top active transcription factors per cell type identified by SCENIC v1.3.1.

Click here to download Table S18

Table S19. Validation of marker genes for each cluster of ***Drosophila*** hemocytes. A list of Gal4-enhancer trap and GFP-exon trap fly lines used for ex vivo validation is included.

Click here to download Table S19

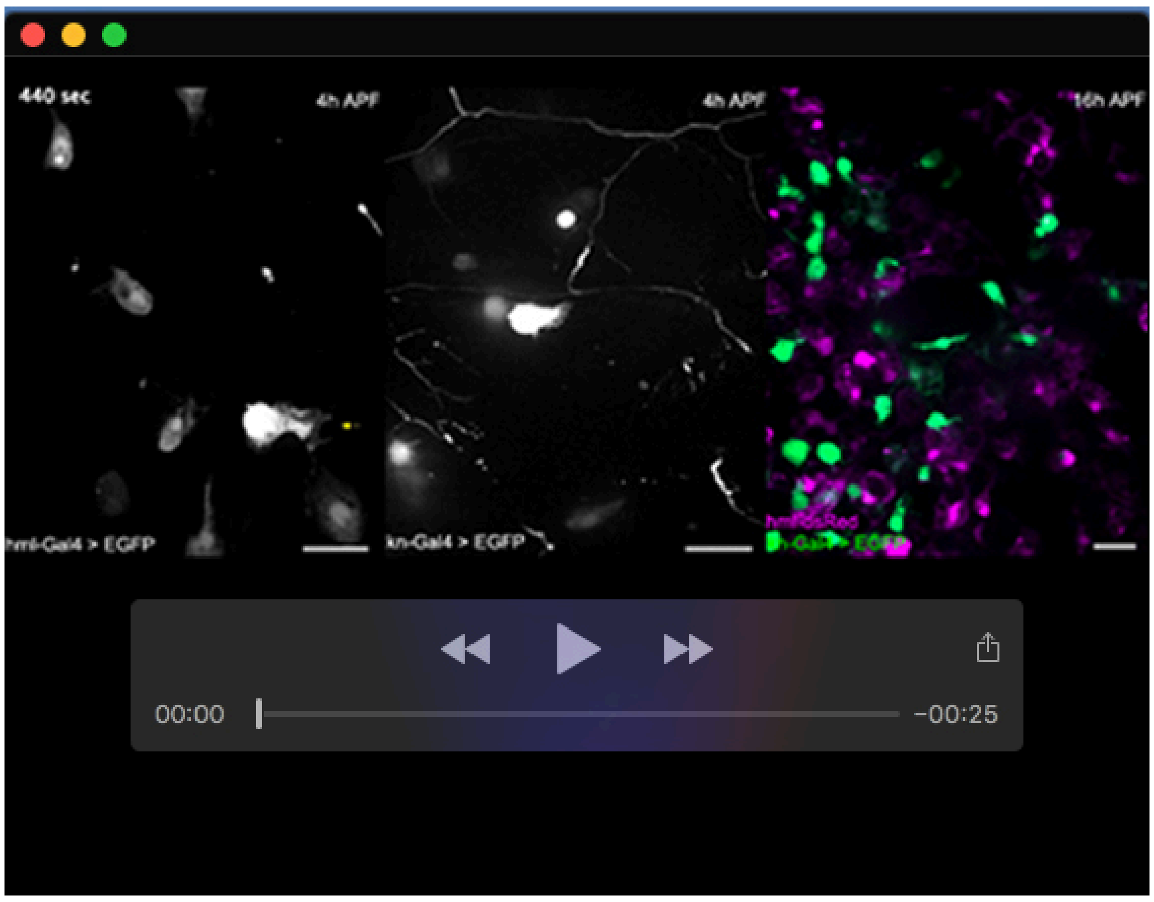

Movie 1. Two different hemocyte populations in pupae marked by *hml* and *kn*. Representative spinning disc microscopy videos of randomly migrating pupal *hml*+ versus *kn*+ cells expressing an EGFP transgene imaged from a living prepupa (4h APF) as indicated. Right: Co-labeling of migrating *kn*+ cells (green) and *hml*+ plasmatocytes (magenta) in a 16h APF old pupa. Scale bar represents 20 µm. Cells were imaged for 30 minutes. Scale bar represents 20 µm.

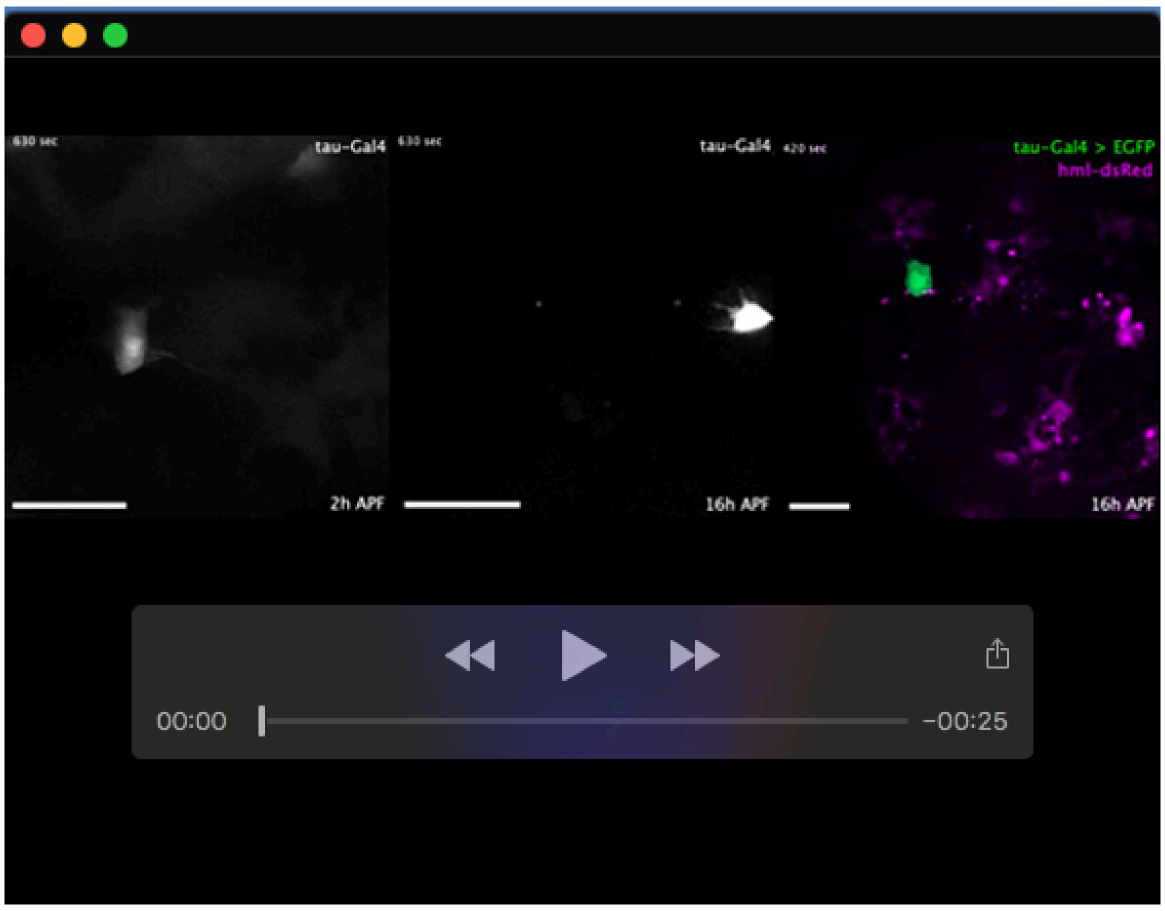

Movie 2. Tau-marked PSC cells are highly migratory. Representative spinning disc microscopy videos of randomly migrating pupal *tau*+ cells expressing an EGFP transgene imaged from a living prepupa at 4h APF (left) and at 16h APF (middle). Right: Co-labeling of a single migrating *kn*+ cells (green) in close contact to *hml*+ plasmatocytes (magenta) in a 16h APF old pupa. Scale bar represents 20 µm.

Development • Supplementary information

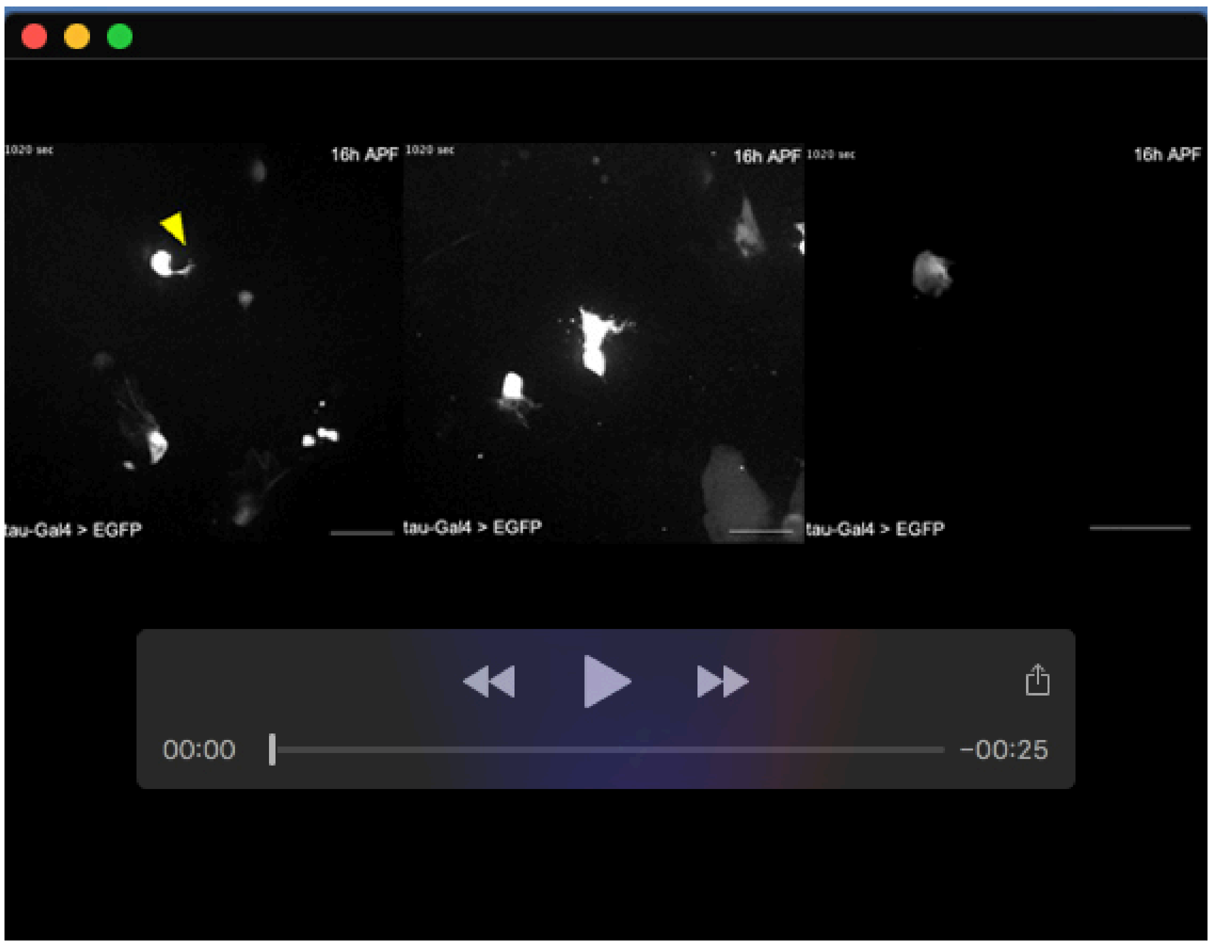

Movie 3. Immune-responsive PSC cells are capable of phagocytosis. Representative spinning disc microscopy videos of migrating pupal *tau* positive cells expressing an EGFP transgene imaged from living pupae (16h APF) upon laser-induced cell ablation (green asterisks). Left: Note the formation of dynamic filopodial protrusion toward the wound. Middle: *tau*-positive PSC cell (yellow arrowhead) starts to phagocytose particles derived from the laser-ablated cell (green asterisk). Right: Single *tau*-positive PSC cell directly migrates towards a laser-ablated cell. Scale bar represents 20 µm.

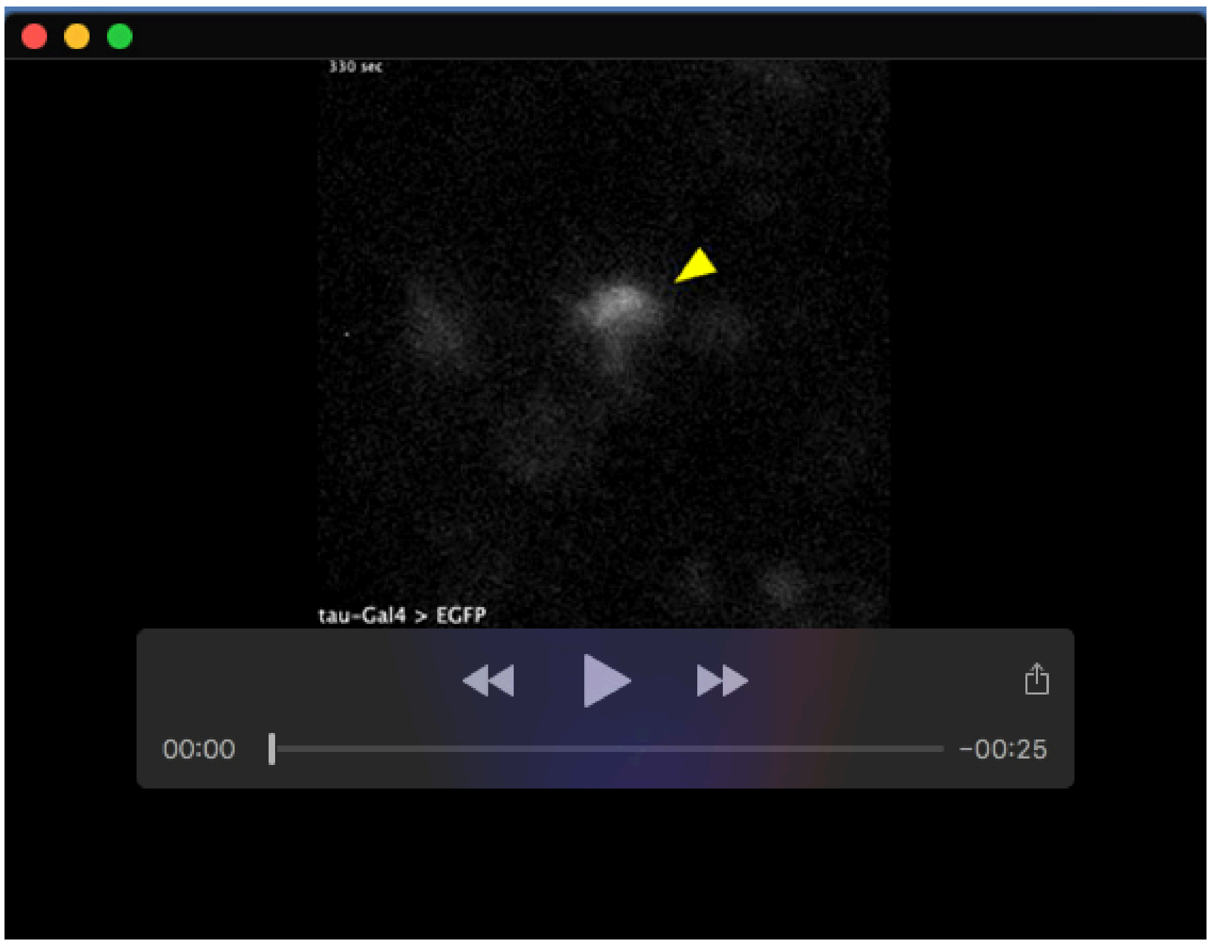

Movie 4. PSC cells have the ability to divide. Single *tau*-positive PSC cell (yellow arrowhead) rounds up and divides. Scale bar represents 20 µm.
