# Supplementary material for: Single-cell transcriptomics identifies new blood cell populations in Drosophila released at the onset of metamorphosis
Source: Development. 2023 Sep 27;150(18):dev201767. doi: 10.1242/dev.201767 (PMC10560556; doi:10.1242/dev.201767)
Supplement: Supplementary file 1 [file develop-150-201767_review_history.pdf]

Single-cell transcriptomics identifies new blood cell populations in *Drosophila* released at the onset of metamorphosis

Alexander Hirschhäuser, Darius Molitor, Gabriela Salinas, Jörg Großhans, Katja Rust and Sven Bogdan
DOI: 10.1242/dev.201767

Editor: Thomas Lecuit

Review timeline

Original submission:	13 March 2023
Editorial decision:	17 April 2023
First revision received:	17 July 2023
Editorial decision:	21 August 2023
Second revision received:	21 August 2023
Accepted:	31 August 2023

Original submission

First decision letter

MS ID#: DEVELOP/2023/201767

MS TITLE: Single-cell transcriptomics identifies new blood cell populations in Drosophila released at the onset of metamorphosis

AUTHORS: Alexander Hirschhaeuser, Darius Molitor, Gabriela Salinas, Joerg Grosshans, Katja Rust, and Sven Bogdan

I have now received all the referees' reports on the above manuscript, and have reached a decision. The referees' comments are appended below, or you can access them online: please go to BenchPress and click on the 'Manuscripts with Decisions' queue in the Author Area.

As you will see, the referees express strong interest in your work, but have some important criticisms and recommend a substantial revision of your manuscript before we can consider publication. If you are able to revise the manuscript along the lines suggested, which may involve further experiments, I will be happy receive a revised version of the manuscript. The comments are quite thorough and constructive. In my view, exploring the functional role of the Osiris-PL cell population is not required for publication at this point (see Rev 1). Your revised paper will be re-reviewed by the original referees, and acceptance of your manuscript will depend on your addressing satisfactorily the reviewers' major concerns. Please also note that Development will normally permit only one round of major revision. If it would be helpful, you are welcome to contact us to discuss your revision in greater detail. Please send us a point-by-point response indicating your plans for addressing the referee's comments, and we will look over this and provide further guidance.

Please attend to all of the reviewers' comments and ensure that you clearly highlight all changes made in the revised manuscript. Please avoid using 'Tracked changes' in Word files as these are lost in PDF conversion. I should be grateful if you would also provide a point-by-point response detailing how you have dealt with the points raised by the reviewers in the 'Response to Reviewers' box. If you do not agree with any of their criticisms or suggestions please explain clearly why this is so.

© 2023. Published by The Company of Biologists under the terms of the Creative Commons Attribution License (https://creativecommons.org/licenses/by/4.0/).

Reviewer 1

Advance summary and potential significance to field

The manuscript by Hirschh¤user et al., reports on hemocyte subpopulations found at the pupal stage. They perform single-cell transcriptomic analysis of cells obtained after bleeding 2-10H old pupae. Several subsets of pupal blood cells have been identified. The authors then focus their analyses on PSC cells, which correspond to niche cells in the lymph gland, the larval hematopoietic organ. Their data establishes that PSC derived cells are migratory and can transdifferentiate into lamellocytes in response to wasp parasitism. However, an essential control is missing to reach this conclusion (see below). The main interest of this manuscript is that it provides the first molecular description of the drosophila blood cells present at the pupal stage. This resource will be valuable for the community. In conclusion, this study is mainly descriptive and since functional analyses are missing, the overall conceptual advances of the study remain limited.

Comments for the author

General comments:
-There are many erroneous statements, references are missing, and some sentences are unclear (see below for details). A more rigorous writing is required.
-This study allows the authors to identify a new hemocyte subpopulation called “Osiris-PL” which might be specifically from pupae. However, the authors did not investigate their function which is an important point to address, since it might be a novel, pupa-specific hemocyte cluster.
-The authors established that PSC cells can give rise to lamellocytes in response to parasitism, but the level of analysis is very limited. The potential significance of PSC cells trans differentiation is not analyzed or discussed.
What is the proportion of PSC cells that transdifferentiate into lamellocytes?
When during development (larval and/or pupal stages) can PSC cells give rise to lamellocytes? Wasp egg encapsulation by lamellocytes takes place at the larval stage; is there any contribution of PSC-derived lamellocytes to the immune response?
Additional comments
-Fig. 4 H-L: Since spiky cells, which the authors attribute to PSC cells, are labelled by tep4 and ance-gal4 drivers, are these two drivers expressed in the PSC of lymph glands in third instar larvae?
-Sup Fig. 6C: Published data indicate that” free larval hemocytes” express kn tau and Antp (Cattenoz et al.,) suggesting that PSC-like cells originate from embryonic hematopoiesis. This point has to be clarified to avoid any misinterpretation. Immunostainings with antibodies against Kn, Antp and Tau should help solve this important point. Establishing the expression pattern using Gal4 drivers might be misleading, since Gal4 drivers might not reproduce the full expression pattern of the corresponding genes.
-Fig 5K: There is a mistake in the labeling. CG31174 and tau have been exchanged.

-Fig 5 L-L”: An important control is missing. Indeed, the expression pattern of tau-Gal4 in response to parasitism has to be established? Is tau-gal4 switched on in LG progenitors in response to parasitism? In this case lamellocytes which are labelled in pupa, in response to parasitism, might result from progenitors and not from PSC cells .
-Fig 6 A-A’, C-C”’ and Fig 7A, D, F and Fig 7 G: Analyzing Kn-GAL4 >EGFP is not informative, since Kn-Gal4 labels a mixed cell population (PSC cells and progenitors from posterior lobes). Thus, Kn-Gal4 >EGFP is redundant with Hml
>GFP analyses shown in Fig 7E. Fig 7A, D, F and Fig 7 G), and movies (M1, M4)
with Kn-Gal4 are useless, since they are redundant and don't add much to the body of results. Thus, they can be deleted.
-Table 1 has to be completed, since for Transitory PL1 and 2; Chitinase PL and Adhesive PL there are some similarities with subsets of hemocytes identified in previous published data
-P4, The reference concerning LG disruption at the pupal stage is missing
(Girgordian et al., 2011). It has to be added on p4 and p20.
-P4, “PSC is dispensable for blood progenitor maintenance “. This sentence is erroneous. Many studies indicate that the PSC controls subsets of lymph gland progenitors. This sentence has to be modified according to published data, and the corresponding references have to be added.

© 2023. Published by The Company of Biologists under the terms of the Creative Commons Attribution License (https://creativecommons.org/licenses/by/4.0/).

-P 6, last paragraph: “Figure 1D supplementary data 17-18”. What does this mean?
-P 16, last paragraph: “12 cell clusters showed significant expression of Serpent”. This is incorrect, since in Fig 1D only 6 subclusters express Srp
(which is in agreement with the text p10, 2nd paragraph).
-P11, last paragraph: “LSP- bomanin-Pl … express all known hemocyte markers including srp “ This is incorrect, srp levels are very low and similar to muscle and neurons (Fig. S1E).
-P7, 1st paragraph: The authors must illustrate crystal cell expression in pupae.

-P7, last paragraph: “differential expression in OxPhos-Pl or Adhesive-Pl and Hydrolysing -PL” and in the Legend of Fig. 3. What are Hydrolysing -PL?
-P7: “dissolved LG” is not a correct term. This has to be modified.

-P7, last sentence: “the upregulation of tau … pupariation”. This sentence in not clear. What do the authors mean?
-P8: “adult flies appear to have hemocytes … has been refuted”. This is not clear. The sentence has to be rephrased.
P9, 1st paragraph: “Consistently, undifferentiated PL expresses genes which encode proteins involved in hematopoiesis”. So what? This sentence is useless.
-P10, 1st line: Sup Fig 3D, E: No data relative to Sup Fig 3D is given in the text.
-Fig S3A : How has the list of the genes analyzed in this figure been stablished
?
-P 10 and Table 1: Osiris Pl has a prolife related to Pl-0 (Cattenoz et al.,),
PM1-2 (Cho et al.,) and PLnAchRalpha3Trol (Li et al.,). Why do the authors consider that they correspond to a new sub-cluster which is specifically from the pupal stage? If this is the case, it is important to address their function.
-P13: chitinase-Pl and adhesive-Pl share similarities with hml+grh (Fig S3A and Li et al.,). Why is this information not indicated in the text and Table 1?
-P13, 2nd paragraph: “dorsal, … were highly expressed in secretory-Pl confirming the importance of this cell type in innate immunity”. This is incorrect, since no data relative to the function of those cells had been established so far.
-P18: “this indicates that Kn positive posterior lobe cells cease to express Kn after dissociation of the lymph gland”. Not OK. kn-Gal4 expression ceased to be expressed but no data relative to endogenous Kn expression is provided.
-P19, last paragraph: “PSC cells can function as tissue resident immune-
responsive cells reservoir with limited phagocytic activity”. This sentence is very misleading: i) since no data support that they can be “resident immune-
cells “, ii) why do the authors talk about limited phagocytic activity, where are the data supporting this statement? iii) the term “reservoir” is not adapted, since in EL3 larval stage each PSC is composed of 20 cells maximum.
This is very few compared to all hemocytes present in larvae or pupae.
-P19, 1st paragraph, last sentence:” This reflects the presence of…. Posterior lobe signaling center”. This sentence in unclear and has to be rephrased.
-P19: “our data … highlight a second new pupal blood cell type derived from the posterior lobe signaling center persisting to adulthood”? This is an overstatement; in this study no data support that Kn positive cells from lymph gland posterior lobes are present in adult!

-P20, last sentence: “Undifferentiated PL appear to not display proliferative precursor stage “. Where are the data supporting this conclusion?
-P21: “Adult flies are devoid of a prohemocyte stage …. that active hematopoiesis ceases in larval stages”. To my knowledge no published data establish that prohemocytes do not exist in adults or that hematopoiesis stops in larvae!

-P21, 2nd paragraph: “which highlights the divers hemocyte functions during pupal development “. This statement is incorrect, since no functional data are provided in this ms.
-P22, last paragraph:” high-resolution live-imaging microscopy analysis using kn-Gal4 and tau-gal4 …”. Once again kn-Gal4 cannot be used to identify PSC cells, since it labels in a mixed cell pool!
-P22, last paragraph: “with distinct molecular signature and cellular functions”. This is incorrect since no data relative to their function are provided in this study.
-In the text and figures legends: Kn>GFP cells cannot be called “PSC cells”,
since Kn-gal4 is expressed in a mixed cell population. This has to be corrected.

© 2023. Published by The Company of Biologists under the terms of the Creative Commons Attribution License (https://creativecommons.org/licenses/by/4.0/).

-Fig 1D: “transcription factor activity”. What does this mean? How is the activity of a transcription factor established?
-Fig 5 A-B and 5 E-F: What are the differences between the A and E, and B and F?

-Fig 4: The title is incorrect, since Secretotry-PL is not a” new plasmatocyte type in pupae” They shared similarities with both larval and lymph gland hemocytes (see the corresponding text and Table 1).
-Fig 5 title: “individual tissue-resident PSC cells”. The title is inappropriate, since no data sustain that there is tissue resident hemocytes.
- In MM a detailed description of wasp parasitism and G-trace experiments is required.

Reviewer 2

Advance summary and potential significance to field

In their manuscript, Bogdan et al. investigate drosophila pupal cell characteristics. Using single-cell RNA sequencing on bleeds from early pupae, they identify various blood cell subpopulations, essentially related to plasmatocyte/macrophage-like cells. Interestingly, their comparative analyses with previously published scRNA-seq data on blood cells from the larval and adult stages indicate that some of these populations exhibit distinct transcriptional signature and seem specific to the pupal stage. In addition, the authors argue that cells derived from the larval lymph gland hematopoietic niche (the Posterior Signaling Center /PSC) are able to differentiate into lamellocytes in response to wasp infection. Using live imaging, they also describe the migratory behavior of PSC-derived cells and show that they can migrate toward wounds.

Globally, the manuscript is clear and well written, with a balanced presentation of the field. This important and timely work provides new insights into the diversity of drosophila blood cells and their evolution across developmental stages. Thus far, the pupal hemocyte repertoire has been poorly characterised and this work significantly extend our knowledge. The bioinformatics and annotation analyses of the scRNAseq data are rather thorough and these data will be a useful resource for researcher in the field. Moreover, the identification of pupal-specific hemocyte populations raises interesting questions concerning their role and it is expected that future studies based on these results will bring new insights into blood cell functional diversification. The possibility that larval hematopoietic niche cells (PSC cells) might differentiate into lamellocytes and participate in the pupal immune response is also interesting and somehow fits with recent findings showing that PSC-derived cells can differentiate into plasmatocyte in the adult.

Yet, several aspects of the manuscript should still be improved to strengthen or validate its conclusions. In particular, the present work suffers from two main drawbacks: (1) the authors do not validate the existence of distinct pupal blood cell populations with additional approaches, (2) the conclusion that PSC-derived cells can give rise to lamellocytes is clearly not supported by sufficient evidence.

Comments for the author

The different points exposed below should be addressed by the authors in a revised manuscript.

Main issues:
(1) The scRNA-seq data analysis supports the idea that pupal hemocyte are heterogenous and can be subdivided into different clusters, some of which seem specific to the pupal stage. However, the authors do not sufficiently use their data to validate in vivo that the combination of some markers they identified can label distinct pupal blood cell populations (for instance undifferentiated PL from AMP-PL or OxPhos-PL); nor do they show that some markers (e.g. for Osiris-PL) are specific to the pupal stage as compared to larval and adult stages. In addition it would be interesting to gain insights into the proportion of these different blood cell types in the pupa (and their evolution at different time of metamorphosis). Along that line, the scRNAseq data are not fully convincing:

© 2023. Published by The Company of Biologists under the terms of the Creative Commons Attribution License (https://creativecommons.org/licenses/by/4.0/).

dataset 2 contributed to around 75% of all the cells and the proportion of the different cluster is very different among the 3 datasets. Unexpectedly too chitinase-PL are is mostly contributed by dataset 1 and 3 (where they represent around 3% of all cells, against only 0.2% in dataset 2). A similar trend is observed for AMP-PL, while Oxphos-PL and PSC are mostly coming from dataset 2. These inconsistencies should at least be discussed and further in vivo validations should be sought. Finally, the authors present a list of GAL4 /GFP lines (suppl data 19) used to “validate” their results but their analysis is not well presented in the text and only a small fraction of the lines tested turned out to label some pupal cells. Overall, the validation of new pupal blood cell markers/populations needs to be improved. The description of their molecular signature could also be shorten.

(2) The authors conclude that PSC cells can differentiate into lamellocytes but the data are not convincing. First, the authors should demonstrate that the “actin-rich” cells that they show Figure 6 are really lamellocytes. Indeed, the morphological criteria generally attributed to lamellocytes do not seem to be met: these actin-rich cells are not particularly larger than the other hemocytes and their nucleus is also of normal size, while differentiated lamellocytes (in the larva at least) are much bigger than normal hemocytes and contain a large endoreplicated nucleus. The authors should at least show that these actin-rich cells express lamellocyte-specific markers such as L1 or myospheroid/ß-integrin for which antibodies are readily available. Second, the authors must demonstrate that tau-GAL4 is specific to the PSC and thus uniquely label PSC-derived hemocytes. Thus far, they only showed that tau-GAL4>UAS-GFP drives specifically in the larval PSC but they have not performed lineage tracing analysis (G-trace) to show that this driver is never expressed in other cells of the larval lymph gland or in circulating larval blood cells, either in normal conditions or following wasp infestation (before lymph gland dispersal). Ideally, the G-trace analysis should also be extended to other tissues because the larval tissues are degraded during metamorphosis and could contribute to “circulating” cells in the pupa.

Finally, in Fig 5K, PSC cells (with spiky morphology) labelled with kn-GAL4 or tau-GAL4 represent 16 or 8% of all the hemocytes whereas in the scRNA-seq, the PSC cluster represent only 0.5% of all the cells. While the authors argue that PSC cells might have been lost during the centrifugation step for scRNA-seq sample preparation, there is only around 100 PSC cells in the lymph gland versus 5.000-10.000 other lymph gland hemocytes and a similar number of circulating larval hemocytes. So one wouldn’t expect PSC cells to account for much more than 0.5% of all the hemocytes in the pupa (unless they proliferate massively in the early pupa). Again, this calls for a better characterization of the “PSC” cluster markers and lineage.

Other issues:
(3) Abstract and main text: the authors do not provide evidence for specific hemocyte populations with distinct “cellular functions”. Actually, their analysis of the specific gene signatures does not really point toward a clear (potential) role for those different populations. Defining the function of these new cell types will be important but could represent a tantalizing project. In the meantime, I recommend that the authors use a more cautious wording.
(4) page 3 : typo: humeral ≠ humoral
(5) page 3: lack of reference for crystal cell function.
(6) page 3: strictly speaking, U-shaped is not a transcription factor (does not bind DNA).
(7) page 4 line 2: the 2x2 references are separated by brackets. And I’m not convinced that Jung et al. as well as Mandal et al. fit here.
(8) Page 4: concerning lymph gland dispersal during pupariation, the work of Grigorian et al. 2011 (doi: 10.1007/s00427-011-0364-6) should be cited.
(9) page 6: typo after Figure 1A.
(10) page 6/Figure S1 : there is a problem of nomenclature correspondence with the main text. I suppose that Precursor-PL1 here corresponds to undifferentiated-
PL and that precursor-PL2, precursor-PL3 stand for transitory-PL1, transitory-PL2.
(11) page 7 and 14: the lack of crystal cells in the scRNAseq and marker analysis is strange. Although they may burst during sample preparation, I’d expect to see some in pupal bleeds as we (not published) can see Bc-GFP or Bc-RFP+ cells through the pupal case.
(12) page 8: the heading “undifferentiated plasmatocyte subtypes with potential to specify” is unclear.
(13) page 12: persephone and spirit should be in italic.
(14) page 13: typo: (Khadilkar et al. 2017) (Williams 2009)

© 2023. Published by The Company of Biologists under the terms of the Creative Commons Attribution License (https://creativecommons.org/licenses/by/4.0/).

(15) page 14 : « confirming the importance of this cell type in innate immunity": this remains to be proven, still hypothetical.
(16) page 17, figure 5K: I guess that CG31174 and tau have been swapped as tau should label only PSC cells and CG31174 only PL morphology cells.
(17) page 17: typo: infestion ≠infection/infestation
(18) page 17: Lebestky et al. 2000 does not fit here- they did not investigate PSC role in stem cell maintenance or lamellocytes induction.
(19) page 18: which kn-GAL4 line was used? Two distinct ones are mentioned in the M&M.
(20) page 18: the heading is misleading; the authors do not show that PSC cells persist in the adult. The same holds true for the conclusion page 19.
(21) page 19 & Figure 7: panels D and E do not correspond to the text description.
(22) page 23: many genes /genotypes are not in italic.
(23) page 26: I didn't see any anti-Atilla staining in the manuscript and Hnt immunostaining is only referred to as "data not shown".
(24) page 26: typo (twice): flour ≠ fluor
(25) page 27 : reference formatting problem : 9, 89 ?
(26) page 34 / Figure 2 legend: typo: transitory PL1-2, -3 ?
(27) page 35-36/Figures 4-5-6 legend: some genes/transgenes are not in italic.

L. Waltzer

Reviewer 3

Advance summary and potential significance to field

In this manuscript entitled "Single-cell transcriptomics identifies new blood cell populations in Drosophila released at the onset of metamorphosis", Hirschh¤user & Molitor, et al. elegantly use single cell transcriptomics approach to map the Drosophila pupal blood cells and identify hemocytes of different molecular signatures. Additionally, this study identifies that the posterior signaling center (PSC), which is known to serve as the lymph gland niche, possesses novel migratory and immune responsive functions. Interestingly, the authors suggest that PSC can also transdifferentiate into Lamellocytes, deeming them as a novel cell type in pupa and adults, besides their roles as the stem cell niche in the larval lymph gland. This study is important to the fly blood cell and hematopoiesis field and will complement the existing blood cell atlases pertaining to larval and adult Drosophila stages.

Comments for the author

Below are a few important and highly recommended suggestions and comments that the authors may want to consider to enhance the quality of this manuscript.

Major Comments:
In the text related to Fig.1, the authors describe the identified clusters very briefly. A systematic and elaborate description of each cluster and why they term them as undifferentiated, transitory, Osiris, etc. must be described right in the beginning (as opposed to later in the results section) for better clarity for the readers. Also, Fig. 1C could be replaced by Fig. 2E.

The authors' naming of the clusters is based on the marker genes that they identified and term them as Spermatid-Marker-PL, Lsp-Bomanin-PL, Osiris-PL, etc. Pupal hemocytes are highly phagocytic and are known to clear tissue debris during metamorphosis. In my opinion, these clusters of PL cells might just be phagocytic PLs that have ingested cells of other tissues. The remnant RNA of extra-PL cell types could therefore be easily captured and detected by scRNA-seq analysis. Can the authors rule this possibility out by validating some of the marker genes (eg., the Osiris group of genes or the spermatid related genes) expressed by the pupal PLs. This is important before claiming certain cell populations (eg. Osiris-PL) as "new cell population" with specialized functions. Real expression vs remnant RNA of other tissues/cell types must be addressed.

© 2023. Published by The Company of Biologists under the terms of the Creative Commons Attribution License (https://creativecommons.org/licenses/by/4.0/).

Same reasoning goes for Spermatid-Marker-PL. What if these PLs are simply PLs that ingested spermatocytes and thereby display most marker genes (and TF modules) of Spermatids? This is important to address as the authors also discuss that this could be an important immune-active PL population (in Discussion). Again, what if these scenarios are due to remnant RNA from the spermatid debris within PLs and captured by scRNA-seq?

Similarly, according to the authors, the Lsp cluster partially resembles fat body cells, but they are not fat body… and PLs in pupae can ingest fat body cells. Hence, validation of a few of these types of marker genes will certainly be helpful to showcase the diverse and “true” expression patterns of PLs.

The tracks in Figure 7D,E are a bit confusing. It is not clear regarding the ‘random’ to ‘directed’ migration based on the figures. Moreover, the lack of depiction of the wound site makes it even more unclear.

Minor comments:
Page 8: Is nAChRalpha3 also called troll? I could not find any synonyms named “troll” for this gene. There are a lot of figure mismatches in text referring to Figure 7. Especially, Figure 7D, E, F. 4 h APF is missing in one of the figures in Fig. 7 D or E. The wound site has to be depicted on Figure 7 D,E. The figure legend for Fig. 7 mentions 4 h and 16 h APF while the actual figures depict 2 h APF. Please double check the entire figure 7 including the corresponding text, figure legend, and the figures.

First revision

Author response to reviewers' comments

Reviewer 1

Advance Summary and Potential Significance to Field:
The manuscript by Hirschhäuser et al., reports on hemocyte subpopulations found at the pupal stage. They perform single-cell transcriptomic analysis of cells obtained after bleeding 2-10H old pupae. Several subsets of pupal blood cells have been identified. The authors then focus their analyses on PSC cells, which correspond to niche cells in the lymph gland, the larval hematopoietic organ. Their data establishes that PSC derived cells are migratory and can transdifferentiate into lamellocytes in response to wasp parasitism. However, an essential control is missing to reach this conclusion (see below). The main interest of this manuscript is that it provides the first molecular description of the drosophila blood cells present at the pupal stage. This resource will be valuable for the community. In conclusion, this study is mainly descriptive and since functional analyses are missing, the overall conceptual advances of the study remain limited.

We agree with the reviewer that we presented the first molecular description of pupal *Drosophila* blood cells. But in contrast, compared to all previous mainly descriptive scRNA studies we also provided first insights into blood cell functional diversification and plasticity, but also how identified cell subtypes actually look like *ex vivo* and *in vivo* (see also below).

Reviewer 1 Comments for the Author
General comments:
-There are many erroneous statements, references are missing, and some sentences are unclear (see below for details). A more rigorous writing is required.

We have substantially restructured our manuscript, particularly we now focused on presenting our data in a more straightforward manner within the context of what has been previously published. We also ensured that our revised manuscript falls within Development's limit of 7000 words (without title page, abstract, materials and methods section and reference list).

© 2023. Published by The Company of Biologists under the terms of the Creative Commons Attribution License (https://creativecommons.org/licenses/by/4.0/).

-This study allows the authors to identify a new hemocyte subpopulation called “Osiris-PL” which might be specifically from pupae. However, the authors did not investigate their function which is an important point to address, since it might be a novel, pupa-specific hemocyte cluster.

As suggested by the reviewers, we could indeed validate two additional pupal cell clusters. First, we identified the Tumor Necrosis Factor (TNF) receptor Grindelwald (Grnd) as a specific marker for the Osiris PL cluster. Immunostainings with a specific anti-Grnd antibody confirmed the differential expression of Grnd in a subset of pupal but not larval plasmatocytes (5,5% positive cells, n=488; new Figure 3A, B). This further supports our findings that Osiris PL cluster represents a novel, pupa-specific hemocyte cluster associated with inflammation.

Secondly, we further identified the Transition protein-like 94D (Tpl94D) encoding a HMG-box domain protein as a good marker for the Spermatid-PL cluster. In immunostainings using an anti-Tpl94D antibody we labeled a subset of hemocyte nuclei accounting for about 10% of total pupal blood cells (new Figure 3C, D).

We agree with the reviewer #1 that the exact role of these cells remains to be investigated, but as reviewer #2 already stated this “tantalizing project” would be a long way beyond the scope of this work.

-The authors established that PSC cells can give rise to lamellocytes in response to parasitism, but the level of analysis is very limited. The potential significance of PSC cells trans differentiation is not analyzed or discussed. What is the proportion of PSC cells that transdifferentiate into lamellocytes?

We thank the reviewer for this suggestion. We further quantified the proportion of PSC cells that transdifferentiate into lamellocytes. In particular, we found that approximately 3.2 % of all Atilla-positive lamellocytes derived from PSC cells upon wasp infestion, whereas the majority of Atilla-positive lamellocytes cells have different origins. We provided this important information in the new figure 6C, D and quantification in figure 6E.

When during development (larval and/or pupal stages) can PSC cells give rise to lamellocytes? Wasp egg encapsulation by lamellocytes takes place at the larval stage.

Flies are regularly attacked by parasitoid wasps of the species Leptopilina boulardi, strain G401, which use their ovipositors to inject eggs into the larvae. If unchecked, wasp larvae hatch approximately 2d post-infection and feed upon host tissues, eventually emerging from and killing the host pupae.

Remarkably, using the G-trace system we could only detect G-traced positive lamellocyten in pupal bleeds but not in the larval hemolymph.

Is there any contribution of PSC-derived lamellocytes to the immune response?

PSC-derived lamellocytes express the lamelocyte marker Atilla and do also not differ morphologically from other lamellocytes in terms of their size and prominent dense actin cytoskeleton. Thus, this suggests that they play the same role in the immune response against wasp infestation.

Additional comments
-Fig. 4 H-L: Since spiky cells, which the authors attribute to PSC cells, are labelled by tep4 and ance-gal4 drivers, are these two drivers expressed in the PSC of lymph glands in third instar larvae?

As recently described in detail, Tep4 is expressed in the MZ of the primary lobes, and in most cells of the secondary lobes and some cell clusters in the tertiary and quaternary lobes (Rodrigues et al. 2021). Ance has been described as a pan progenitor marker expressed in different lymph gland regions (Kanwal et al. 2021). We confirmed such a broad expression of Ance in the primary, secondary and tertiary lobes, but decided not to show it due to unnecessary redundancy.

© 2023. Published by The Company of Biologists under the terms of the Creative Commons Attribution License (https://creativecommons.org/licenses/by/4.0/).

Please note that we also showed that spiky cells are positive for tau, which we cannot detect outside the PSC niche in the lymph gland (Fig. 5L). In agreement, we can detect Tep4 and Ance in spiky cells as well as cells with regular plasmatocyte morphology (Fig. 4 J,L) while tau exclusively labels spiky cells (Fig. 5L).

-Sup Fig. 6C: Published data indicate that" free larval hemocytes" express kn, tau and Antp (Cattenoz et al.,) suggesting that PSC-like cells originate from embryonic hematopoiesis. This point has to be clarified to avoid any misinterpretation. Immunostainings with antibodies against Kn, Antp and Tau should help solve this important point. Establishing the expression pattern using Gal4 drivers might be misleading, since Gal4 drivers might not reproduce the full expression pattern of the corresponding genes.

As suggested by the reviewer we further quantified the number of *kn*- and *tau*-positive cells in larvae and pupae (see new figure 5K). Immunostainings with an antibody against Kn exactly correspond with the *kn*-Gal4 driver expression. As already mentioned in our first manuscript version, *kn* also labeled cells with a more plasmatocyte-like round morphology (new Figure 5K). *Kn*-positive cells make up 15.2% of the pupal hemocytes (n= 636), but only 4.4% of the cells show a spiky shape (new Figure 5K). Interestingly, we also found *kn*-positive cells in larval bleed (3,4 %, n=351; new figure 5K). However, all labeled larval cells showed a plasmatocyte-like round morphology suggesting that these morphologically different cells have distinct origins (new Figure 5K). Since *kn* is not only expressed in the lymph gland PSC but also in the MZ and posterior lobes (Benmimoun et al., 2015b; Boulet et al., 2021; Kanwal et al., 2021; Rodrigues et al., 2021), additional *kn*-positive cells with plasmatocyte morphology might derive from the MZ or posterior lobes.

Unfortunately, the only available DSHB antibody against recombinant human Tau protein (with confirmed species reactivity for *Drosophila*, 5A6) did not work in immunostainings. However, the tau-Gal4 driver exclusively labeled small spiky cells from pupal bleeds but in a lower overall number (1.4 %, n= 573; new Figure 5K). No *tau* positive hemocytes could be found in larva (n = 505; new figure 5K).

-Fig 5K: There is a mistake in the labeling. CG31174 and tau have been exchanged.

We apologize for this and changed it.

-Fig 5 L-L": An important control is missing. Indeed, the expression pattern of tau-Gal4 in response to parasitism has to be established? Is tau-gal4 switched on in LG progenitors in response to parasitism? In this case lamellocytes which are labelled in pupa, in response to parasitism, might result from progenitors and not from PSC cells.

We thank the reviewer and we addressed this important issue. Importantly, we could not detect tau-Gal4 activity outside the PSC niche in the lymph gland (compare also new Figure 5L-L'). We could also not detect any additional lymph gland tau expression even upon parasitoid wasp infection. Instead, we observed a progressively reduced number of tau-positive cells which goes along an earlier lymph gland dispersal induced by wasp infection (new Figure 6C, C', D, D'; quantification in new figure 6E, E').

-Fig 6 A-A', C-C"' and Fig 7A, D, F and Fig 7 G: Analyzing Kn-GAL4 >EGFP is not informative, since Kn-Gal4 labels a mixed cell population (PSC cells and progenitors from posterior lobes). Thus, Kn-Gal4 >EGFP is redundant with Hml >GFP analyses shown in Fig 7E. Fig 7A, D, F and Fig 7 G), and movies (M1, M4) with Kn-Gal4 are useless, since they are redundant and don't add much to the body of results. Thus, they can be deleted.

We agree that kn-Gal4 labels a mixed cell population and we removed all kn-Gal4 data in figure 6 as suggested by the reviewer. However, we still included a new supplementary movie (M1) showing a *kn*-positive hemocyte population co-labeled with hml-dsRed positive plasmatocytes. Indeed, *kn* and *hml* label two distinct populations. To our knowledge, this shows for the first time a migrating hemocyte subpopulation which is distinct but in close contact to classical hml-positive plasmatocytes in a living pupa.

© 2023. Published by The Company of Biologists under the terms of the Creative Commons Attribution License (https://creativecommons.org/licenses/by/4.0/).

In addition, we provided new images of *tau*-marked cells in a new figure 7 and new supplementary movies. These new data sets further strengthen our findings that 1. PSC cells are highly motile and immune-responsive cells with limited phagocytic activity (new quantification in figure 7E, new supplementary movies M2, M3). 2. Co-labeling experiments further revealed a close contact of rare PSC cells with highly abundant plasmatocytes (new figure 7C and supplementary movie M2), interacting by multiple filopodial protrusions reminiscent of lymph gland PSC cells (Krzemien et al., 2007; Mandal et al., 2007).

-Table 1 has to be completed, since for Transitory PL1 and 2; Chitinase PL and Adhesive PL there are some similarities with subsets of hemocytes identified in previous published data.

We thank the reviewer and we changed this accordingly.

-P4, The reference concerning LG disruption at the pupal stage is missing (Girgordian et al., 2011). It has to be added on p4 and p20.

We apologize for this missing reference and we included it.

-P4, “PSC is dispensable for blood progenitor maintenance “. This sentence is erroneous. Many studies indicate that the PSC controls subsets of lymph gland progenitors. This sentence has to be modified according to published data, and the corresponding references have to be added.

We apologize for this erroneous sentence and replaced it as follows “The PSC was initially suggested to control the balance between undifferentiated precursor cells and differentiating hemocytes (Krzemien et al., 2007; Mandal et al., 2007). However, more recent studies further revealed a more complex role of the PSC in regulating blood cell differentiation, rather than maintaining core progenitors in lymph gland homeostasis (Benmimoun et al., 2015a; Benmimoun et al., 2015b; Oyallon et al., 2016).

-P 6, last paragraph: “Figure 1D supplementary data 17-18”. What does this mean?

We apologize for any confusion. Supplementary data 17-18 include the original data set used for the SCENIC analysis. Figure 1D shows the transcription factor activity of selected transcription factors identified by SCENIC.

-P 16, last paragraph: “12 cell clusters showed significant expression of Serpent”. This is incorrect, since in Fig 1D only 6 subclusters express Srp (which is in agreement with the text p10, 2nd paragraph).

We corrected this mistake and added the following sentence in the paper: “In the pupa, we found several cell clusters with higher levels of *srp* expression and *srp* activity (SCENIC), including undifferentiated PL, transitory PL1-2, Osiris-PL and Secretory-PL (Figure 1D, 2A-B). Expression analysis of a *srp* Gal4 promoter trap that we expect to reflect *srp* mRNA levels confirmed that *srp* expression is heterogeneous in hemocytes (Figure 2C).”

-P11, last paragraph: “LSP- bomanin-Pl … express all known hemocyte markers including srp “This is incorrect, srp levels are very low and similar to muscle and neurons (Fig. S1E).

We agree with the reviewer and removed this statement.

-P7, 1st paragraph: The authors must illustrate crystal cell expression in pupae.

As suggested, we further quantified the number of Hnt-positive crystal cells in larvae and pupae. Accordingly, we only found very few Hnt-positive hemocytes isolated from pupal hemolymph (0.37 %, n= 545) compared to larval preparations (3.48 %, n= 517). This suggests that crystal cells represent either a rare cell type in Drosophila pupae and adults as recently reported (Ghosh et al., 2015), or many cells may have been lost due to the sensitive nature of crystal cells, which tend to burst after bleeding (Bidla et al., 2007; Hultmark and Ando, 2022). We included these new data in text (see page 7).

© 2023. Published by The Company of Biologists under the terms of the Creative Commons Attribution License (https://creativecommons.org/licenses/by/4.0/).

-P7, last paragraph: “differential expression in OxPhos-Pl or Adhesive-Pl and Hydrolysing -PL” and in the Legend of Fig. 3. What are Hydrolysing -PL?

We apologize for any confusion. We replaced “Hydrolysing -PL” with “Chitinase-PL”.

-P7: “dissolved LG” is not a correct term. This has to be modified.

We thank the reviewer and removed this term.

-P7, last sentence: “the upregulation of tau … pupariation”. This sentence in not clear. What do the authors mean?

We thank the reviewer and removed this sentence.

-P8: “adult flies appear to have hemocytes … has been refuted”. This is not clear. The sentence has to be rephrased.

We thank the reviewer and removed this sentence.

P9, 1st paragraph: “Consistently, undifferentiated PL expresses genes which encode proteins involved in hematopoiesis”. So what? This sentence is useless.

We thank the reviewer and removed this sentence.

-P10, 1st line: Sup Fig 3D, E: No data relative to Sup Fig 3D is given in the text

We apologize for any confusion and removed it.

-Fig S3A : How has the list of the genes analyzed in this figure been stablished?

We assessed the expression of all genes published as hemocyte subtype specific markers. Markers that displayed cluster specific expression in our dataset are useful for the comparison of pupal clusters with hemocyte subtypes identified in other stages and are thus shown in this Table. We also modified the description of Figure S3A: “Dotplot of the average and percent expression of hemocyte subtype specific markers identified in other studies (Cattenoz et al., 2020, Cho et al., ,2020, Li et al., 2022, Fu et al., 2020, Tattikota et al., 2020, Girard et al., 2021). This table includes only markers that display cluster specific expression in our dataset.”

-P 10 and Table 1: Osiris Pl has a prolife related to Pl-0 (Cattenoz et al.,), PM1-2 (Cho et al.,) and PLnAchRalpha3Trol (Li et al.,). Why do the authors consider that they correspond to a new sub-cluster which is specifically from the pupal stage? If this is the case, it is important to address their function.

We apologize if our previous writing was mistakable and added two statements in the text that clarify that undifferentiated-PL correspond to these clusters while Osiris-PL does not have a corresponding cluster: P7: “This cluster [undifferentiated-PL] corresponds to a larval plasmatocyte population (PL-0, Cattenoz et al., 2020; PLASM1, Leitao et al., 2020) and/or a lymph gland plasmatocyte population (PM1-2, Cho et al., 2020; PL2, Girard et al., 2021) as well as an adult plasmatocyte subtype (plasmatocytes nAChRalpha3 and *trol* high, Li et al., 2022; supplementary figure S3A, supplementary table 1).”

P9: “Interestingly, Osiris-PL did not express a gene profile that correlates to larval, lymph gland or adult cell population suggesting that it might be a new cell population of specialized hemocytes specific for metamorphosis (see Supplementary Figure S3A, supplementary table 1).”

-P13: chitinase-Pl and adhesive-Pl share similarities with hml+grh (Fig S3A and Li et al.,). Why is this information not indicated in the text and Table 1

We thank the reviewer for noticing this error. Supplementary table 1 previously noted the similarity between Adhesive-PL and hml+ with grh and we now also note the similarity with Chitinase-PL.

© 2023. Published by The Company of Biologists under the terms of the Creative Commons Attribution License (https://creativecommons.org/licenses/by/4.0/).

-P13, 2nd paragraph: “dorsal, … were highly expressed in secretory-Pl, confirming the importance of this cell type in innate immunity”. This is incorrect, since no data relative to the function of those cells had been established so far.

We agree and removed the sentence.

-P18: “this indicates that Kn positive posterior lobe cells cease to express Kn after dissociation of the lymph gland”. Not OK. kn-Gal4 expression ceased to be expressed but no data relative to endogenous Kn expression is provided.

We agree and removed the sentence.

-P19, last paragraph: “PSC cells can function as tissue resident immune-responsive cells reservoir with limited phagocytic activity”. This sentence is very misleading: i) since no data support that they can be “resident immune-cells “,

We agree and removed the term “resident”.

ii) why do the authors talk about limited phagocytic activity, where are the data supporting this statement?

As suggested by the reviewer, we now provide new data supporting this notion. We isolated tau-positive cells by FACS and performed a phagocytosis assay. These new data confirmed a limited phagocytosis activity compared to professional phagocytic hml-marked plasmatocytes (new Figure 7E).

iii) the term “reservoir” is not adapted, since in EL3 larval stage each PSC is composed of 20 cells maximum. This is very few compared to all hemocytes present in larvae or pupae.

We agree and removed the term “reservoir”.

-P19, 1st paragraph, last sentence:” This reflects the presence of…. Posterior lobe signaling center”. This sentence in unclear and has to be rephrased.

We agree and removed the sentence.

-P19: “our data … highlight a second new pupal blood cell type derived from the posterior lobe signaling center persisting to adulthood”? This is an overstatement; in this study no data support that Kn positive cells from lymph gland posterior lobes are present in adult!

We isolated hemocytes from the adult hemolymph indicating that both kn-positive cells but also tau-positive cells indeed persist in adults. Confocal image of kn- and tau-positive cells isolated from the adult hemolymph is shown in the new (Figure 6J, K).

-P20, last sentence: “Undifferentiated PL appear to not display proliferative precursor stage “. Where are the data supporting this conclusion?

This statement is not present in the current version of the manuscript anymore as we had to shorten the text due to space limitations. The associated data supporting this statement was a bioinformatic analysis of cell cycle specific gene expression that was previously present in Figure S3A (previous submission).

-P21: “Adult flies are devoid of a prohemocyte stage …. that active hematopoiesis ceases in larval stages”. To my knowledge no published data establish that prohemocytes do not exist in adults or that hematopoiesis stops in larvae!

We agree and removed the sentence.

© 2023. Published by The Company of Biologists under the terms of the Creative Commons Attribution License (https://creativecommons.org/licenses/by/4.0/).

-P21, 2nd paragraph: "which highlights the divers hemocyte functions during pupal development ". This statement is incorrect, since no functional data are provided in this ms.

We agree and removed the sentence.

-P22, last paragraph:" high-resolution live-imaging microscopy analysis using kn-Gal4 and tau-gal4 …". Once again kn-Gal4 cannot be used to identify PSC cells, since it labels in a mixed cell pool!

We agree and removed the sentence. As mentioned above, we provided new images of tau-marked cells in a new figure 7 and new supplementary movies. These new data sets further strengthen our findings that 1. PSC cells are highly motile and immune-responsive cells with limited phagocytic activity (new quantification in figure 7E, new supplementary movies M2, M3). 2. Co-labeling experiments further revealed a close contact of rare PSC cells with highly abundant plasmatocytes (new figure 7C and supplementary movie M2), interacting by multiple filopodial protrusions reminiscent of lymph gland PSC cells (Krzemien et al., 2007; Mandal et al., 2007).

-P22, last paragraph: "with distinct molecular signature and cellular functions". This is incorrect since no data relative to their function are provided in this study.

We agree and replaced it by the phrases such as "… hemocytes with different molecular signatures associated to distinct functions …"

-In the text and figures legends: Kn>GFP cells cannot be called "PSC cells", since Kn-gal4 is expressed in a mixed cell population. This has to be corrected.

We agree and removed this as described above.

-Fig 1D: "transcription factor activity". What does this mean? How is the activity of a transcription factor established?

Transcription factor activity is determined by assessing the expression of the transcription factor itself and target genes which contain the known binding site for the transcription factor. If the transcription factor and many target genes are expressed, the transcription factor is likely active. If only few target genes are expressed, the transcription factor is likely inactive or not a major determinant of the transcriptome of the cell. If the transcription factor is not expressed, the activity is not scored. The method is described in depth in the original publication of the SCENIC algorithm (Aibar et al., 2017). We now also cite the paper in the figure legend.

-Fig 5 A-B and 5 E-F: What are the differences between the A and E, and B and F?

Figures 5A and B show the expression levels of kn and Antp respectively. Figures E and F show the activity of kn and Antp. In order to assess the activity, we also consider the expression of known target genes of these transcription factors.

-Fig 4: The title is incorrect, since Secretotry-PL is not a" new plasmatocyte type in pupae" They shared similarities with both larval and lymph gland hemocytes (see the corresponding text and Table 1).

We agree and changed the title of Figure 4 to "Secretory-PL are transcriptomically distinct from other pupal blood cell types".

-Fig 5 title: "individual tissue-resident PSC cells". The title is inappropriate, since no data sustain that there is tissue resident hemocytes.

We agree and removed the term "tissue resident", see above.

- In MM a detailed description of wasp parasitism and G-trace experiments is required.

We apologize and provided a better description.

© 2023. Published by The Company of Biologists under the terms of the Creative Commons Attribution License (https://creativecommons.org/licenses/by/4.0/).

Reviewer 2
Advance Summary and Potential Significance to Field:
In their manuscript, Bogdan et al. investigate drosophila pupal cell characteristics. Using single-cell RNA sequencing on bleeds from early pupae, they identify various blood cell subpopulations, essentially related to plasmatocyte/macrophage-like cells. Interestingly, their comparative analyses with previously published scRNA-seq data on blood cells from the larval and adult stages indicate that some of these populations exhibit distinct transcriptional signature and seem specific to the pupal stage. In addition, the authors argue that cells derived from the larval lymph gland hematopoietic niche (the Posterior Signaling Center /PSC) are able to differentiate into lamellocytes in response to wasp infection. Using live imaging, they also describe the migratory behavior of PSC-derived cells and show that they can migrate toward wounds.

Globally, the manuscript is clear and well written, with a balanced presentation of the field. This important and timely work provides new insights into the diversity of drosophila blood cells and their evolution across developmental stages. Thus far, the pupal hemocyte repertoire has been poorly characterised and this work significantly extend our knowledge. The bioinformatics and annotation analyses of the scRNAseq data are rather thorough and these data will be a useful resource for researcher in the field. Moreover, the identification of pupal-specific hemocyte populations raises interesting questions concerning their role and it is expected that future studies based on these results will bring new insights into blood cell functional diversification. The possibility that larval hematopoietic niche cells (PSC cells) might differentiate into lamellocytes and participate in the pupal immune response is also interesting and somehow fits with recent findings showing that PSC-derived cells can differentiate into plasmatocyte in the adult.

We thank the reviewer for these very positive and constructive comments.

Yet, several aspects of the manuscript should still be improved to strengthen or validate its conclusions. In particular, the present work suffers from two main drawbacks: (1) the authors do not validate the existence of distinct pupal blood cell populations with additional approaches.

We thank the reviewer for this suggestion. Thus, we further validated distinct pupal blood cell populations using additional marker-specific antibodies. In immunostainings we could indeed validate two additional pupal cell clusters. First, we identified the Tumor Necrosis Factor (TNF) receptor Grindelwald (Grnd) as a specific marker for the Osiris PL cluster. Immunostainings with a specific anti-Grnd antibody confirmed the differential expression of Grnd in a subset of pupal but not larval plasmatocytes (5,5% positive cells, n=488; new Figure 3A, B). This further supports our findings that Osiris PL cluster represents a novel, pupa-specific hemocyte cluster associated with inflammation. Secondly, we further identified the Transition protein-like 94D (Tpl94D) encoding an HMG-box domain protein as a good marker for the Spermatid-PL cluster. In immunostainings using an anti-Tpl94D antibody we labeled a subset of hemocyte nuclei accounting for about 10% of total pupal blood cells (new Figure 3C, D).

2) the conclusion that PSC-derived cells can give rise to lamellocytes is clearly not supported by sufficient evidence.

As suggested by the reviewer, we could further strengthen our conclusion that PSC can give rise to lamellocytes. These new data sets include not only new anti-Atilla immunostainings, new live cell imaging but also new quantifications, addressing the proportion of PSC cells that transdifferentiate into lamellocytes and the specificity of the tau-Gal4 driver as a PSC marker.

Reviewer 2 Comments for the Author:
The different points exposed below should be addressed by the authors in a revised manuscript.

Main issues:
(1)The scRNA-seq data analysis supports the idea that pupal hemocyte are heterogenous and can be subdivided into different clusters, some of which seem specific to the pupal stage. However, the authors do not sufficiently use their data to validate in vivo that the combination of some markers they identified can label distinct pupal blood cell populations (for instance undifferentiated PL

© 2023. Published by The Company of Biologists under the terms of the Creative Commons Attribution License (https://creativecommons.org/licenses/by/4.0/).

from AMP-PL or OxPhos-PL); nor do they show that some markers (e.g. for Osiris-PL) are specific to the pupal stage as compared to larval and adult stages.

As mentioned above, we could indeed validate two additional pupal cell clusters. First, we identified the Tumor Necrosis Factor (TNF) receptor Grindelwald (Grnd) as a specific marker for the Osiris PL cluster. Immunostainings with a specific anti-Grnd antibody confirmed the differential expression of Grnd in a subset of pupal but not larval plasmatocytes (5,5% positive cells, n=488; new Figure 3A, B). This further supports our findings that Osiris PL cluster represents a novel, pupa-specific hemocyte cluster associated with inflammation. Secondly, we further identified the Transition protein-like 94D (Tpl94D) encoding an HMG-box domain protein as a good marker for the Spermatid-PL cluster. In immunostainings using an anti-Tpl94D antibody we labeled a subset of hemocyte nuclei accounting for about 10% of total pupal blood cells (new Figure 3C, D).

In addition, it would be interesting to gain insights into the proportion of these different blood cell types in the pupa (and their evolution at different time of metamorphosis). Along that line, the scRNAseq data are not fully convincing: dataset 2 contributed to around 75% of all the cells and the proportion of the different cluster is very different among the 3 datasets. Unexpectedly too, chitinase-PL are is mostly contributed by dataset 1 and 3 (where they represent around 3% of all cells, against only 0.2% in dataset 2). A similar trend is observed for AMP-PL, while Oxphos-PL and PSC are mostly coming from dataset 2. These inconsistencies should at least be discussed and further in vivo validations should be sought.

The datasets have different cell numbers as the two smaller ones were part of a trial series when we optimized the protocol. As all datasets can be successfully integrated (using batch correction) the different cell numbers should not present an issue. Note that for single cell data, every cell in itself is a replicate.

The different contributions to some of the clusters are indeed surprising. We can only speculate why these differences present themselves but we do occasionally see slight differences between preparations. These could be due to different efficiencies during bleeds or because the blood cell populations adjust to the environmental situation. While we seek to raise our flies in an optimal and germ-free environment, it is possible that some vials have more exposure to mold or bacteria. We note in the paper: "All three datasets contributed to each of these 14 clusters, although we noted unequal contribution in some cases (Supplementary figure S1B-D)."

Finally, the authors present a list of GAL4 /GFP lines (suppl data 19) used to "validate" their results but their analysis is not well presented in the text and only a small fraction of the lines tested turned out to label some pupal cells. Overall, the validation of new pupal blood cell markers/populations needs to be improved. The description of their molecular signature could also be shorten.

We have substantially restructured our manuscript and the main text was shortened by more than 2000 words, particularly we now focused on presenting our data in a more straightforward manner within the context of what has been previously published. We also now better presented supplementary data 19.

(2) The authors conclude that PSC cells can differentiate into lamellocytes, but the data are not convincing. First, the authors should demonstrate that the "actin-rich" cells that they show Figure 6 are really lamellocytes. Indeed, the morphological criteria generally attributed to lamellocytes do not seem to be met: these actin-rich cells are not particularly larger than the other hemocytes and their nucleus is also of normal size, while differentiated lamellocytes (in the larva at least) are much bigger than normal hemocytes and contain a large endoreplicated nucleus. The authors should at least show that these actin-rich cells express lamellocyte-specific markers such as L1 or myospheroid/ß-integrin for which antibodies are readily available.

We thank the reviewer for this suggestion. We now included additional anti-Atilla immunostainings indicating that these G-traced actin-rich cells are indeed lamellocytes. These confocal images are included in new figure 6D.

© 2023. Published by The Company of Biologists under the terms of the Creative Commons Attribution License (https://creativecommons.org/licenses/by/4.0/).

Second, the authors must demonstrate that tau-GAL4 is specific to the PSC and thus uniquely label PSC-derived hemocytes. Thus far, they only showed that tau-GAL4>UAS-GFP drives specifically in the larval PSC but they have not performed lineage tracing analysis (G-trace) to show that this driver is never expressed in other cells of the larval lymph gland or in circulating larval blood cells, either in normal conditions or following wasp infestation (before lymph gland dispersal).

We thank the reviewer for this suggestion. Activation of G-trace with *tau*-Gal4 results in labeling of PCS cells (RFP+/GFP+) of third instar lymph gland. No expression can be observed in other cells outside the PSC (Figure 6B-B’’’). We could also not detect any additional lymph gland tau expression even upon parasitoid wasp infection. Instead, we observed a progressively reduced number of tau-positive cells which goes along an earlier lymph gland dispersal induced by wasp infection (new Figure 6C, C’, D, D’; quantification in new figure 6E, E’). In contrast to kn, the tau-Gal4 driver exclusively labeled small spiky cells from pupal bleeds but in a lower overall number (1.4 %, n= 573; Figure 5). No tau positive hemocytes could be found in larva (n = 505; figure 5K).

Ideally, the G-trace analysis should also be extended to other tissues because the larval tissues are degraded during metamorphosis and could contribute to “circulating” cells in the pupa.

As mentioned above, we could not detect tau-Gal4 activity in other cells circulating in the prepupal hemolymph except PSC cells. A G-trace analysis extended to other tissues would be interesting but would be beyond the scope of this work.

Finally, in Fig 5K, PSC cells (with spiky morphology) labelled with kn-GAL4 or tau-GAL4 represent 16 or 8% of all the hemocytes whereas in the scRNA-seq, the PSC cluster represent only 0.5% of all the cells.

We agree with the reviewer. We therefore extended our quantification of *kn*-positive and *tau*-positive cells in pupae, this time from a pool of 2-10 h old pupae, the exact starting material for our scRNA analyses. In these new cell counts, we confirmed that the tau-Gal4 driver exclusively labeled small spiky cells from pupal bleeds but in a lower overall number (1.4 %, n= 573; Figure 5). No *tau* positive hemocytes could be found in larva (n = 505; figure 5K). In contrast, *kn*-positive cells make up 15.2% of the pupal hemocytes (n= 636), but only 4.4% of the cells show a spiky shape (Figure 5K). Interestingly, we also found *kn*-positive cells in larval bleed (3,4 %, n=351; figure 5K). However, all labeled larval cells showed a plasmatocyte-like round morphology suggesting that these morphologically different cells have distinct origins (Figure 5K).

While the authors argue that PSC cells might have been lost during the centrifugation step for scRNA-seq sample preparation, there is only around 100 PSC cells in the lymph gland versus 5.000-10.000 other lymph gland hemocytes and a similar number of circulating larval hemocytes. So one wouldn’t expect PSC cells to account for much more than 0.5% of all the hemocytes in the pupa (unless they proliferate massively in the early pupa). Again, this calls for a better characterization of the “PSC” cluster, markers and lineage.

As mentioned above our new cell counts revealed that *tau*-Gal4 labels a subset of hemocyte nuclei accounting for about 1.4% of total blood cells. Live-imaging analysis and immunostainings using anti-Phospho-Histone H3 antibody did not reveal that PSC proliferate massively in the early pupa, but rather a limited proliferative capacity. Of course, we cannot exclude that PSC cells might have been lost during the centrifugation step.

Other issues:

(3) Abstract and main text: the authors do not provide evidence for specific hemocyte populations with distinct “cellular functions”. Actually, their analysis of the specific gene signatures does not really point toward a clear (potential) role for those different populations. Defining the function of these new cell types will be important but could represent a tantalizing project. In the meantime, I recommend that the authors use a more cautious wording.

As mentioned above, we agree with the reviewers and replaced it by a more cautious wording as follows “… hemocytes with different molecular signatures associated to distinct functions …”

© 2023. Published by The Company of Biologists under the terms of the Creative Commons Attribution License (https://creativecommons.org/licenses/by/4.0/).

(4) page 3 : typo: humeral ≠ humoral

We changed this.

(5) page 3: lack of reference for crystal cell function.

We included the two references: (Bidla et al., 2007; Rizki and Rizki, 1959).

(6) page 3: strictly speaking, U-shaped is not a transcription factor (does not bind DNA).

We changed this: "…and the friend of GATA (FOG) transcriptional cofactor U-shaped (Crozatier and Vincent, 2011)."

(7) page 4 line 2: the 2x2 references are separated by brackets. And I'm not convinced that Jung et al. as well as Mandal et al. fit here.
We changed the references.

(8) Page 4: concerning lymph gland dispersal during pupariation, the work of Grigorian et al. 2011 (doi: 10.1007/s00427-011-0364-6) should be cited.

We included this reference.

(9) page 6: typo after Figure 1A.

We changed this.

(10) page 6/Figure S1 : there is a problem of nomenclature correspondence with the main text. I suppose that Precursor-PL1 here corresponds to undifferentiated-PL and that precursor-PL2, precursor-PL3 stand for transitory-PL1, transitory-PL2.

We apologize for this confusion and changed this.

(11) page 7 and 14: the lack of crystal cells in the scRNAseq and marker analysis is strange. Although they may burst during sample preparation, I'd expect to see some in pupal bleeds as we (not published) can see Bc-GFP or Bc-RFP+ cells through the pupal case.

We agree with the reviewer and we therefore reexamined this. We indeed confirmed a very low number of crystal cells in pupae. We only found very few Hnt-positive hemocytes isolated from pupal hemolymph (0.37 %, n= 545) compared to larval preparations (3.48 %, n= 517). This suggests that crystal cells represent either a rare cell type in *Drosophila* pupae and adults as recently reported (Ghosh et al., 2015), or many cells may have been lost due to the sensitive nature of crystal cells, which tend to burst after bleeding (Bidla et al., 2007; Hultmark and Ando, 2022). We included this additional information in the main text (see page 7).

(12) page 8: the heading "undifferentiated plasmatocyte subtypes with potential to specify" is unclear.

We agree with the reviewer and removed the sentence.

(13) page 12: persephone and spirit should be in italic.

We changed this.

(14) page 13: typo: (Khadilkar et al. 2017) (Williams 2009)

We have removed these references as part of the text shortening.

(15)page 14 : « confirming the importance of this cell type in innate immunity": this remains to be proven, still hypothetical.

© 2023. Published by The Company of Biologists under the terms of the Creative Commons Attribution License (https://creativecommons.org/licenses/by/4.0/).

We have removed these references as part of the text shortening.

(16) page 17, figure 5K: I guess that CG31174 and tau have been swapped as tau should label only PSC cells and CG31174 only PL morphology cells.

We apologize for this confusion and changed it.

(17) page 17: typo: infestion ≠infection/infestation

We apologize for this confusion and changed it.

(18) page 17: Lebestky et al. 2000 does not fit here - they did not investigate PSC role in stem cell maintenance or lamellocytes induction.

We changed it.

(19) page 18: which kn-GAL4 line was used? Two distinct ones are mentioned in the M&M.

We used the kn-Gal4 driver: y[1] w[*]; Mi{Trojan-GAL4.2}kn[MI15480-TG4.2]/SM6a;; (BL 67516) from the Bloomington stock center. The pcol85-Gal4 driver from Crozatier et al., 2004 was removed in the M&M.

(20) page 18: the heading is misleading; the authors do not show that PSC cells persist in the adult. The same holds true for the conclusion page 19.

We isolated hemocytes from the adult hemolymph indicating that both kn-positive cells but also tau-positive cells indeed persist in adults. A confocal image of a tau-positive cells isolated from the adult hemolymph is shown in the new figure 6F.

(21) page 19 & Figure 7: panels D and E do not correspond to the text description.

Thanks, we changed it.

(22) page 23: many genes /genotypes are not in italic.

We changed it.

(23) page 26: I didn't see any anti-Atilla staining in the manuscript and Hnt immunostaining is only referred to as "data not shown".

We thank the reviewer for this suggestion. We now included additional anti-Atilla immunostainings indicating that these G-traced actin-rich cells are indeed lamellocytes. These confocal images are included in new figure 6C.

As mentioned above, we further quantified the number of Hnt-positive crystal cells in larvae and pupae. Accordingly, we only found very few Hnt-positive hemocytes isolated from pupal hemolymph (0.37 %, n= 545) compared to larval preparations (3.48 %, n= 517).

(24) page 26: typo (twice): flour ≠ fluor

We changed it.

(25) page 27 : reference formatting problem : 9, 89 ?

We changed it.

(26) page 34 / Figure 2 legend: typo: transitory PL1-2, -3 ?

We changed it.

© 2023. Published by The Company of Biologists under the terms of the Creative Commons Attribution License (https://creativecommons.org/licenses/by/4.0/).

(27) page 35-36/Figures 4-5-6 legend: some genes/transgenes are not in italic.

We changed it.

L. Waltzer

Reviewer 3
Advance Summary and Potential Significance to Field:
In this manuscript entitled “Single-cell transcriptomics identifies new blood cell populations in Drosophila released at the onset of metamorphosis”, Hirschhäuser & Molitor, et al. elegantly use single cell transcriptomics approach to map the Drosophila pupal blood cells and identify hemocytes of different molecular signatures. Additionally, this study identifies that the posterior signaling center (PSC), which is known to serve as the lymph gland niche, possesses novel migratory and immune responsive functions. Interestingly, the authors suggest that PSC can also transdifferentiate into Lamellocytes, deeming them as a novel cell type in pupa and adults, besides their roles as the stem cell niche in the larval lymph gland. This study is important to the fly blood cell and hematopoiesis field and will complement the existing blood cell atlases pertaining to larval and adult Drosophila stages.

We thank the reviewer for these very positive and constructive comments.

Reviewer 3 Comments for the Author:
Below are a few important and highly recommended suggestions and comments that the authors may want to consider to enhance the quality of this manuscript.

Major Comments:
In the text related to Fig.1, the authors describe the identified clusters very briefly. A systematic and elaborate description of each cluster and why they term them as undifferentiated, transitory, Osiris, etc. must be described right in the beginning (as opposed to later in the results section) for better clarity for the readers.

We have substantially restructured our manuscript and the main the text was shortened by more than 2000 words, particularly we now focused on presenting our data (including the description of the cluster) in a more straightforward manner within the context of what has been previously published.

Also, Fig. 1C could be replaced by Fig. 2E.

Figure 1C aims to show that each cluster we identified is transcriptomically distinct. We used the top 50 differentially expressed genes for each cluster for this heatmap. Figure 2E concentrates on a subset of clusters (undifferentiated PL, transitory PL1-2 and Osiris PL) and shows 20 distinct markers each which helped us to determine a potential function of each cell type.

The authors’ naming of the clusters is based on the marker genes that they identified and term them as Spermatid-Marker-PL, Lsp-Bomanin-PL, Osiris-PL, etc. Pupal hemocytes are highly phagocytic and are known to clear tissue debris during metamorphosis. In my opinion, these clusters of PL cells might just be phagocytic PLs that have ingested cells of other tissues. **The remnant RNA of extra-PL cell types could therefore be easily captured and detected by scRNA-seq analysis.**

We considered this possibility and performed an extensive literature research. Neither single cell transcriptomics analyses assessing Drosophila blood cells (Cattenoz et al., 2020, Cho et al., ,2020, Li et al., 2022, Fu et al., 2020, Tattikota et al., 2020, Girard et al., 2021) nor human blood cells (Tabula Sapiens Consortium* et al. 2022; Domínguez Conde et al. 2022) have reported this issue.
In addition, publications have shown that mRNA decay occurs very early on during apoptosis, prior to membrane lipid scrambling, DNA fragmentation and, importantly, prior to phagocytosis (Thomas et al., 2015 (http://dx.doi.org/10.1016/ j.celrep.2015.04.026), Bedoui et al., 2020 (https://doi.org/10.1038/s41580-020-0270-8)). It is therefore unlikely that mRNA of phagocytosed material is intact enough to be sequenced.

© 2023. Published by The Company of Biologists under the terms of the Creative Commons Attribution License (https://creativecommons.org/licenses/by/4.0/).

Can the authors rule this possibility out by validating some of the marker genes (eg., the Osiris group of genes or the spermatid related genes) expressed by the pupal PLs.

As suggested by the reviewers, we could indeed validate these two pupal cell clusters. First, we identified the Tumor Necrosis Factor (TNF) receptor Grindelwald (Grnd) as a specific marker for the Osiris PL cluster. Immunostainings with a specific anti-Grnd antibody confirmed the differential expression of Grnd in a subset of pupal but not larval plasmatocytes (5,5% positive cells, n=488; new Figure 3A, B). This further supports our findings that Osiris PL cluster represents a novel, pupa-specific hemocyte cluster associated with inflammation. Secondly, we further identified the Transition protein-like 94D (Tpl94D) encoding an HMG-box domain protein as a good marker for the Spermatid-PL cluster. In immunostainings using an anti-Tpl94D antibody we labeled a subset of hemocyte nuclei accounting for about 10% of total pupal blood cells (new Figure 3C, D).

This is important before claiming certain cell populations (eg. Osiris-PL) as “new cell population” with specialized functions. Real expression vs remnant RNA of other tissues/cell types must be addressed. Same reasoning goes for Spermatid-Marker-PL. What if these PLs are simply PLs that ingested spermatocytes and thereby display most marker genes (and TF modules) of Spermatids?

As mentioned above, we now provide additional immunostainings validating this cell cluster.

This is important to address as the authors also discuss that this could be an important immune-active PL population (in Discussion). Again, what if these scenarios are due to remnant RNA from the spermatid debris within PLs and captured by scRNA-seq?

As mentioned above, we could indeed validate two additional pupal cell clusters, the Osiris-PL and the Spermatid-PL.

Similarly, according to the authors, the Lsp cluster partially resembles fat body cells, but they are not fat body… and PLs in pupae can ingest fat body cells. Hence, validation of a few of these types of marker genes will certainly be helpful to showcase the diverse and “true” expression patterns of PLs.

As mentioned above, it appears unlikely that mRNA of phagocytosed fat body is stable enough to be detected by RNA sequencing. Bioinformatically, Lsp-Bomanin-PL express many gene also expressed in fat body. However, fat body cells are polyploid and have therefore large nuclei. Using our ICELL8 data we were able to determine that Lsp-Bomanin-PL nuclei are small and similar in size to other plasmatocyte subtypes.

The tracks in Figure 7D,E are a bit confusing. It is not clear regarding the ‘random’ to ‘directed’ migration based on the figures. Moreover, the lack of depiction of the wound site makes it even more unclear.

As suggested by reviewer #1 kn-Gal4 labels a mixed cell population and we thus removed all kn-Gal4 data in figure 6 as suggested by the reviewer. For all new ablation experiments of tau-marked cells we clearly marked the wound site by a green asterisk.

Minor comments:
Page 8: Is nAChRalpha3 also called troll? I could not find any synonyms named “troll” for this gene.

This adult hemocyte cluster was named according to two genes that are highly expressed: nAChRalpha3 and trol. To avoid confusion, we now write the entire cluster name: “plasmatocytes nAChRalpha3 and trol high”.

There are a lot of figure mismatches in text referring to Figure 7. Especially, Figure 7D, E, F. 4 h APF is missing in one of the figures in Fig. 7 D or E. The wound site has to be depicted on Figure 7 D, E. The figure legend for Fig. 7 mentions 4 h and 16 h APF while the actual figures depict 2 h APF. Please double check the entire figure 7 including the corresponding text, figure legend, and the figures.

© 2023. Published by The Company of Biologists under the terms of the Creative Commons Attribution License (https://creativecommons.org/licenses/by/4.0/).

We apologize for the confusion and changed it.

Second decision letter

MS ID#: DEVELOP/2023/201767

MS TITLE: Single-cell transcriptomics identifies new blood cell populations in Drosophila released at the onset of metamorphosis

AUTHORS: Alexander Hirschhaeuser, Darius Molitor, Gabriela Salinas, Joerg Grosshans, Katja Rust, and Sven Bogdan

I have now received all the referees reports on the above manuscript, and have reached a decision. The referees' comments are appended below, or you can access them online: please go to BenchPress and click on the 'Manuscripts with Decisions' queue in the Author Area.

The overall evaluation is very positive and we would like to publish your manuscript in Development. Before we can proceed, could you please address the text edits suggested by the reviewers.

Reviewer 2

Advance summary and potential significance to field

The revised manuscript provides valuable insights into pupal blood cell diversity and PSC cell plasticity. It also integrates previous scNRA_seq results on larval and adult blood cells to bring a rather comprehensive comparison of drosophila blood cell populations at different stages of development. This is a timely report
which open some new lines of investigations concerning the functional importance of these different blood cell types.

Comments for the author

The authors made a good job at revising their manuscript and they have addressed my main concerns. In particular:
- they validated by immunostainings the expression of 2 hemocyte subtype markers (although double/combinatorial immunostainings would have been better suited to establish that their expression is restricted/enriched in a particular population rather than just stochastic).
- they performed lineage tracing experiments in the lymph gland with tau-GAL4 to demonstrate that it is solely expressed in PSC cells. If possible, the image displayed in Fig 6B should be changed for a more convincing one: even though tau-GAL4 live/traced expression is restricted to cells at the posterior part of the anterior lobes, these cells do not form a typical PSC-like structure as observed for instance in Fig 6E.
The authors have also corrected many errors and streamlined their analysis, making the manuscript more rigorous and agreeable to read.
Besides a few typos which can be rectified at the proofreading stage, I'd like to suggest two corrections:
(1) concerning the role of the PSC (page 4): the work of Baldeosingh et al.
2018 (PMID 29966604) showing that the PSC controls a subpopulation of progenitors could be cited together with Benmimoun and Oyallon publications.
(2) concerning the quote that Srp "was recently proposed as a progenitor marker in adult fly 'Ghosh et al. 2015" (page 7). This assumption of Ghosh et al. 2015 was proven wrong by Sanchez-Bosch et al. 2019 (PMID 31735669). The paragraph should be modified accordingly to better reflect the current literature (as it was the case in their original version of the manuscript).
L. Waltzer

© 2023. Published by The Company of Biologists under the terms of the Creative Commons Attribution License (https://creativecommons.org/licenses/by/4.0/).

Reviewer 3

Advance summary and potential significance to field

In this revised manuscript Hirschhäuser & Molitor, et al. thoroughly re-investigated and satisfactorily addressed all the questions and all recommended changes proposed have been considered by the authors. The manuscript has been re-written well and substantially improved.

Comments for the author

Below are some final minor corrections that need attention:
Several areas including Abstract, G-trace should be changed to G-TRACE (all caps as it is an abbreviation), including in main figures (Fig. 6A-E). Although both versions have been used in the paper, for consistency, it should be G-TRACE.

Not sure if there exists an abbreviations page, but certain abbreviations must be introduced/expanded somewhere. Examples: AMP-PL is first introduced on Page#8 but AMP is not expanded anywhere. It must be shown as Antimicrobial peptide (AMP)-PL at least once...So is for PL, that it refers to Plasmatocyte.

In Figure 2C', "srpHemo-3XmCherry" within the micrograph could be changed to gray to match the color of the cells.

Second revision

Author response to reviewers' comments

Reviewer 2 Comments for the Author:

The authors made a good job at revising their manuscript and they have addressed my main concerns. In particular:
- they validated by immunostainings the expression of 2 hemocyte subtype markers (although double/combinatorial immunostainings would have been better suited to establish that their expression is restricted/enriched in a particular population rather than just stochastic).

We thank the reviewer for these very positive and constructive comments. Combinatorial immunostainings with compatible immunosera will be indeed a valuable tool for our future work.

- they performed lineage tracing experiments in the lymph gland with tau-GAL4 to demonstrate that it is solely expressed in PSC cells. If possible, the image displayed in Fig 6B should be changed for a more convincing one: even though tau-GAL4 live/traced expression is restricted to cells at the posterior part of the anterior lobes, these cells do not form a typical PSC-like structure as observed for instance in Fig 6E.

We would like to keep the image displayed in Fig 6B.

The authors have also corrected many errors and streamlined their analysis, making the manuscript more rigorous and agreeable to read.

Besides a few typos which can be rectified at the proofreading stage, I'd like to suggest two corrections:
(1) concerning the role of the PSC (page 4): the work of Baldeosingh et al. 2018 (PMID 29966604) showing that the PSC controls a subpopulation of progenitors could be cited together with Benmimoun and Oyallon publications.

© 2023. Published by The Company of Biologists under the terms of the Creative Commons Attribution License (https://creativecommons.org/licenses/by/4.0/).

As suggested by the reviewer we further included this reference in the manuscript.

(2) concerning the quote that Srp "was recently proposed as a progenitor marker in adult fly 'Ghosh et al. 2015" (page 7). This assumption of Ghosh et al.

2015 was proven wrong by Sanchez-Bosch et al. 2019 (PMID 31735669). The paragraph should be modified accordingly to better reflect the current literature (as it was the case in their original version of the manuscript).

As suggested by the reviewer we reincluded the paragraph including the reference from the original version of the manuscript, as follows:
"The GATA transcription factor Serpent (Srp), which is required for prohemocyte specification in the embryo (Rehorn et al., 1996), was previously proposed as progenitor marker in the adult fly (Ghosh et al., 2015), however, the presence of a proliferating hemocyte precursor stage in adults has been refuted more recently (Sanchez Bosch et al., 2019)."

Reviewer 3
Advance Summary and Potential Significance to Field:
In this revised manuscript, Hirschhäuser & Molitor, et al. thoroughly re- investigated and satisfactorily addressed all the questions and all recommended changes proposed have been considered by the authors. The manuscript has been re- written well and substantially improved.

Reviewer 3 Comments for the Author:
Below are some final minor corrections that need attention:

Several areas including Abstract, G-trace should be changed to **G-TRACE** (all caps, as it is an abbreviation), including in main figures (Fig. 6A-E). Although both versions have been used in the paper, for consistency, it should be G-TRACE.

We changed this in the manuscript text and in figure 6.

Not sure if there exists an abbreviations page, but certain abbreviations must be introduced/expanded somewhere. Examples: **AMP**-PL is first introduced on Page#8, but AMP is not expanded anywhere. It must be shown as Antimicrobial peptide (AMP)-PL at least once...So is for PL, that it refers to Plasmatocyte.

We now introduced AMP abbreviation on page 11 of the manuscript text, as follow: "*Among the immunity genes we identified a large repertoire of AMP (Antimicrobial peptide) encoding genes like Attacin-A, Attacin-B, Drosomycin-like 1, Drosomycin-like 6, Cecropin A, Cecropin B and Drosocin (Figure 3K).*"

In Figure 2C', "**srpHemo-3XmCherry**" within the micrograph could be changed to gray to match the color of the cells.

We changed this in figure 2C.

Third decision letter

MS ID#: DEVELOP/2023/201767

MS TITLE: Single-cell transcriptomics identifies new blood cell populations in Drosophila released at the onset of metamorphosis

© 2023. Published by The Company of Biologists under the terms of the Creative Commons Attribution License (https://creativecommons.org/licenses/by/4.0/).

AUTHORS: Alexander Hirschhaeuser, Darius Molitor, Gabriela Salinas, Joerg Grosshans, Katja Rust, and Sven Bogdan
ARTICLE TYPE: Research Article

I am happy to tell you that your manuscript has been accepted for publication in Development, pending our standard ethics checks.

© 2023. Published by The Company of Biologists under the terms of the Creative Commons Attribution License (https://creativecommons.org/licenses/by/4.0/).
